# Scalable Fabrication of Metallic Conductive Fibers from Rheological Tunable Semi-Liquid Metals

**DOI:** 10.34133/2022/9890686

**Published:** 2022-10-27

**Authors:** Shujun Tian, Hao Peng, Huaizhi Liu, Jiancheng Zhou, Jiuyang Zhang

**Affiliations:** School of Chemistry and Chemical Engineering, Jiangsu Hi-Tech Key Laboratory for Biomedical Research, Southeast University, Nanjing 211189, China

## Abstract

Conductive polymer fibers/wires (CPFs) are important materials in modern technologies due to their unique one-dimension geometry, electrical conductivity, and flexibility. However, the advanced applications of current CPFs are limited by their low electrical conductivities (<500 S/m) and poor interfacial interactions between conductive fillers (e.g., graphite) and polymers. Therefore, in current electrical applications, metal wires/foils like copper and aluminum are the most frequently utilized conductive fibers/wires instead of the inferior conductive CPFs. This work successfully addresses the heavy phase segregation between polymers and conductive inorganic materials to obtain semiliquid metal polymer fibers (SLMPFs) which exhibit an ultrahigh electrical conductivity (over 10^6^ S/m), remarkable thermal processability, and considerable mechanical performance (Young's modulus: ~300 MPa). Semiliquid metal (gallium-tin alloy) with tunable viscosities is the key to achieve the excellent miscibility between metals and polymers. Both the rheological results and numerical simulations demonstrate the critical viscosity matching for the successful preparation of the fibers. More importantly, the fibers are adapted with classic polymer melt-processing like melt injection, which indicates the scalable production of the highly conductive fibers. The SLMPFs are highly promising substitutes for metal wires/fibers in modern electrical applications such as electricity transmission, data communication, and underwater works.

## 1. Introduction

In recent years, conductive polymer fibers (CPFs) are increasingly important for applications in artificial intelligence, flexible sensors, and optical fiber [1–6] because of their merits of electrical conductivity, low density, and extensive adaptability. Compared to traditional electrical wires (such as copper and aluminum), CPFs have mild fabrication conditions, stretchability, and corrosion resistance which show great potential in the modern intelligent technology [7–9]. The most common fabrication strategy for CPFs is the integration of polymer and inorganic conductive fillers by plastic molding, which forms one-dimension composites that combine the processibility of the polymer matrix and the electrical conductivity of the inorganic dispersion phase [10–12]. The inorganic conductive fillers mainly include carbon-based fillers (graphene, carbon nanotubes, carbon black, etc.) [13–15] and metal fillers [16, 17]. However, due to the intrinsically inferior conductivity of carbon fillers (usually less than 1000 S/m) [18], the electrical conductivities of most carbon-based polymer fibers are always lower than 500 S/m [8, 19, 20]. Besides, carbon-based fillers will dramatically increase the viscosity of composites and cause degradation during the melt-processing [15]. These challenging issues heavily restrict the advanced applications of the fibers in fiber-optic cable, intelligent sensors, and bionics. Different from carbon-based fillers, metals with the superior electrical conductivity are promising candidates for the fabrication of highly conductive CPFs [21–23]. However, metals and polymers have heavy phase separation during processing owing to the notoriously weak interfacial interaction between metals and polymers [24], resulting in undesirable electrical conductivity and mechanical performance. Although silver nanowires (AgNWs) with high aspects provide high conductivity with CPFs, AgNW-based fibers are unsuitable for scalable production because of the high cost of AgNWs and poor stability [25].

Semiliquid metals (SLMs) with unique properties of tunable rheology [12, 17, 21, 26–28] and metallic conductivity [6, 12, 29–33] have been extensively applied in the field of the automotive industry, electronic devices, and aeronautics. With the coexistence of liquid and solid, the fluidity of SLMs can be conveniently mediated by alloy composition and processing conditions based on the classic alloy phase diagram [9, 32, 34–36]. Therefore, the utilization of SLMs may be a possible way to overcome the phase segregation between the polymers and metals due to the unique tunable rheological features of the liquid-solid structures in SLMs.

This work reports a universal strategy to combine gallium-tin alloy (GaSn) and polymers (such as polylactic acid, PLA; thermoplastic polyurethane, TPU) for successfully realizing semiliquid metal polymer fibers (SLMPFs) with superior electrical conductivity (over 10^6^ S/m), excellent thermal processability, and considerable mechanical performance (Young's modulus: ~300 MPa). The high conductivity of SLMPFs is attributed to the viscosity matching between SLM and PLA which effectively eliminates the phase segregation between fillers and polymer matrix. The validity and reliability of rheological matching are demonstrated by rheological experiments, which are highly consistent with numerical simulation results. More importantly, the SLMPFs comply with scalable production methods such as the classic polymer injection, which successfully inherits the excellent processability of polymers even at high SLM loading (55%). Due to the high electrical conductivity, low density, and flexibility, the SLMPFs emerge as an exciting substitute for copper wires in the field of electricity transmission, data communication, and underwater works.

## 2. Results

### 2.1. Fabrication of the Highly Conductive SLMPFs

**Figure 1:**
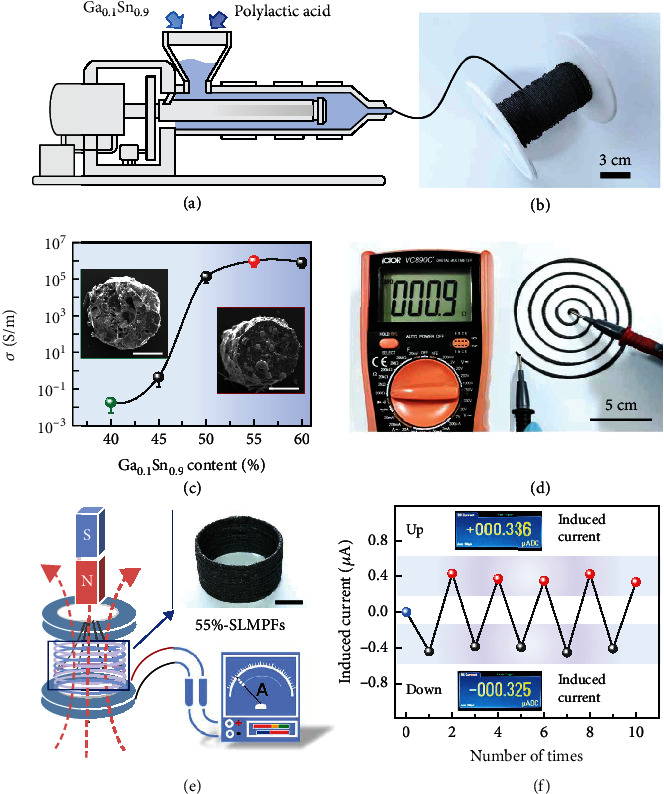
Fabrication of the highly conductive SLMPFs. (a) The fabrication of the semiliquid metal polymer fibers (SLMPFs). (b) A coil twined from 55%-SLMPF (55% is the volume fraction of the metal in the fiber) to show the scalable production. The whole length of SLMPF is more than 20 m. (c) The volume conductivity (*σ*) of SLMPFs with different content of SLM. Insets are the cross-sectional morphologies of 40%-SLMPF and 55%-SLMPF, respectively (scale bar: 200 *μ*m). (d) The resistance of the spiral circuit printed by 55%-SLMPF (size: 10 cm × 10 cm, line diameter: 1.12 mm) is low to 0.9 *Ω* through a simple two-wires measurement. (e) Schematic illustration of electromagnetic induction via the SLMPFs. Insert is the 55%-SLMPFs coil (scale bar: 3 cm). (f) The induced current is generated inside 55%-SLMPF when the magnet moves up and down in the SLMPFs coil. Insets show the value of positive and negative current induced by electromagnetic induction.

Injection is a classic plastic molding technique, which can conveniently prepare polymer fibers with different diameters in a large scale [9, 31, 34, 36].  Figure 1(a) shows the fabrication process and morphology of the SLMPFs. For molding the SLMPFs, the Ga_0.1_Sn_0.9_ alloy powder (average diameter: 100 *μ*m) and polymer (polylactic acid, PLA 3260HP, NatureWorks, Mw = 10800, PDI = 1.61) powder are mixed and injected at 180°C to obtain SLMPFs with various diameters from 200 *μ*m to 20 mm (detailed process in experimental section and Figure S1). Here, we use Ga_x_Sn_y_ to represent the SLM (*x* and *y* note the mole fraction of Ga and Sn, respectively). X%-SLMPF is used to denote the SLMPFs with X% volume fraction of SLM. Despite the high loading, the 55%-SLMPFs maintain the excellent thermal processability inherited from PLA. As shown in Figure 1(b), a 55%-SLMPF whose length exceeds 20 m is continuously collected to a coil (coil diameter: 25 mm), showing the scalable production of SLMPFs via the melt injection. Besides the fibers, SLMPFs are also easily molded to arbitrary forms as desired, such as concentric circle, pyramid, and equilateral triangle (Figure S2).

As shown in Figure 1(c), the conductivity of SLMPFs is significantly determined by the loading of SLM. As increasing SLM content from 40% to 55%, the conductivity of SLMPFs gradually rises from 10^−1^  to 10^6^ S/m, which is across seven orders of magnitude and reaches the metallic conductivity. The conductivity of SLMPFs dramatically increases between 45% and 50%. Insets in Figure 1(c) and EDS mapping (Figure S3) exhibit the cross-sectional morphologies of 40%-SLMPF and 55%-SLMPF with an average diameter of 380 *μ*m, showing the uniform dispersion in micrometer scale of SLM in PLA even at such high loading. As shown in Figure S4, the liquid phase exists around the solid phase which represents the gallium will be limited by the tin and will not leak as the temperature rises. Further increasing the SLM content will not increase the conductivity of SLMPFs but lead to the undesirable aggregation of SLMs in the polymer matrix (Figure S5). This conductivity change of SLMPFs is well consistent with the percolation threshold theory of conductive composites [37]. To visually observe the highly electrical conductivity of 55%-SLMPF, the resistance value of a spiral circuit (size: 10 cm × 10 cm, line diameter: 1.12 mm) printed from 55%-SLMPF (Figure 1(d)) is low to 0.9 *Ω* through a simple two-wires measurement, showing a superior electrical conductivity of 55%-SLMPFs. Besides, the highly conductive 55%-SLMPF can generate the induced current under a changing magnetic field, which is similar with the traditional metal wires (Figure 1(e)).  Figure 1(f) and Movie S1 record the values of induced current with a magnet moving in the 55%-SLMPF coil. When the magnet moves up and down in the coil, the coil generates positive and negative current (~ ±350 *μ*A), respectively. Besides PLA, thermoplastic polyurethane (TPU 1190A, BASF SE, Mw = 10000)-based SLMPFs were successfully fabricated. The uniform miscibility between SLM and TPU is invesitaged by the cross-sectional microscope images of a single 55%-SLMPF (TPU matrix) from different heights (Figure S6), demonstrating the universal validity of this method.

### 2.2. The Properties of SLMPFs

**Figure 2:**
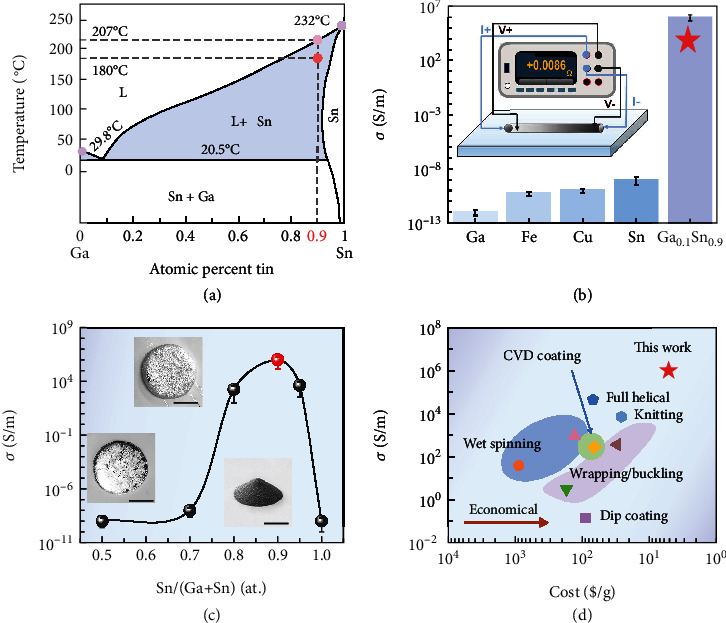
The properties of SLMPFs. (a) The gallium-tin binary alloy phase diagram. The dotted lines mark the composition (Ga_0.1_Sn_0.9_), processing temperature (180°C), and liquidus temperature (207°C) utilized in this work. (b) Volume conductivities of PLA fibers fabricated from different fillers with identical content of 55%. Inset illustrates a 55%-SLMPF whose resistance is low to 0.0086 *Ω* through a four-wire measurement. (c) The volume conductivity (*σ*) of 55%-SLMPFs changes with different compositions of SLM (Ga_x_Sn_y_). Insets are the morphology of the filler Ga_x_Sn_y_ with different atomic ratios at 180°C (scale bar: 2 cm). (d) Comparison of the volume conductivity (*σ*) and economic cost (U.S. dollars/g) of the SLMPFs and previously reported conductive polymer composites, such as full helical [38], knitting [39], chemical vapor deposition coating [40], dip coating [41], wet-spinning [42, 43], and wrapping/buckling [20, 44].

SLM is critical for the fabrication of highly conductive SLMPFs. The key to achieve metallic conductivity of SLMPFs is the SLM with tunable viscosity which can match the viscosity of polymer matrix during processing. The viscosity matching between SLM and polymer means that the SLM will flow along with PLA at a similar velocity during shear mixing. In other words, the relative movement velocity between SLM and polymer is near zero during processing. Hence, viscosity matching will bring a relatively uniform distribution of SLM in PLA compared with giant differences on viscosity between pure metals and PLA (Figure S7). Remarkably different from the pure metal, the gallium-tin phase diagram (Figure 2(a)) shows multiple phase transitions of SLM: below 20.5°C (i.e., solidus), the SLM (Ga_0.1_Sn_0.9_) is in the solid state; when the temperature is higher than 20.8°C, Ga_0.1_Sn_0.9_ transfers into solid-liquid state and maintains the semiliquid state till 207°C; beyond 207°C (i.e., liquidus), the Ga_0.1_Sn_0.9_ becomes full liquid (Figure S8). Moreover, the stable alloy composition (Ga_0.1_Sn_0.9_) has been verified by the cyclic DSC test (Figure S9) and isothermal experiments (Figure S10), and no obviously phase separation overserved. The stable alloy composition ensures the reliability of rheological test. The SLMPF also inherits such thermal stability of SLM and avoids the leakage of SLM, which was confirmed by the isothermal experiments (Figure S11). As shown in Figure 2(b), fibers incorporated with different fillers at an identical content of 55 vol% were fabricated by the same method as preparing SLMPFs (details are shown in the experimental section). The pure metal powders (Figure S12) have a tendency to be simply wrapped by polymer matrix rather than interconnected to construct the conductive network (Figure S13), causing the volume conductivities of the composites (55%Fe-PLA, 55%Cu-PLA, and 55%Sn-PLA) are lower than 10^−8^ S/m. In addition, the Ga cannot be mixed into PLA matrix to achieve electrically conductive since gallium is in a complete liquid state. As shown in Figure S14, massive Ga flowed out from PLA. In sharp contrast, SLMs (Ga_x_Sn_y_) offer opportunities for SLMPFs to reach high electrical conductivity over 10^6^ S/m (GaSn-PLA). Inset illustrates that a 55%-SLMPF was measured by a multimeter in four-wire modes: the resistance can reach as low as 0.0086 *Ω*, showing a superior electrical conductivity of 55%-SLMPFs. Furthermore, the composition of Ga_x_Sn_y_ also affects the volume conductivity of SLMPFs at room temperature (Figure 2(c)). The conductivity of SLMPFs gradually increases from 10^−9^ to 10^6^ S/m as the atomic ratio of gallium to tin changes from 5 : 5 to 1 : 9. Further increment of the atomic ratio of Sn scarifies the volume conductivity (10^−8^ S/m) of SLMPFs because the Ga_0.05_Sn_0.95_ in polymer matrix severely aggregates (Figure S15). Insets in Figure 2(c) show the morphological differences of SLM particles with different atomic ratios at 180°C, indicating that the phase state of SLM is heavily influenced by their chemical composition.

The processing conditions also dominate the performance of SLMPFs. Figure 2(d) summarizes the volume conductivity and economic cost (U.S. dollars/g) of previously reported CPFs to make a comparison with SLMPFs. Such ultrahigh conductivity (10^6^ S/m) as well as low cost is among the best of the previously reported CPFs such as full helical [38], knitting [39], chemical vapor deposition coating [40], dip coating [41], wet-spinning [42, 43], and wrapping/buckling [20, 44]. The traditional CPFs usually have limited electrical conductivities from 10^1^ to 10^4^ S/m due to the inferior conductivity of the conductive fillers. Benefiting from the high-effective injection technique, the production of SLMPFs is far more economic than most previously reported CPFs which are fabricated by complicated techniques.

### 2.3. The Processing Conditions of SLMPFs

**Figure 3:**
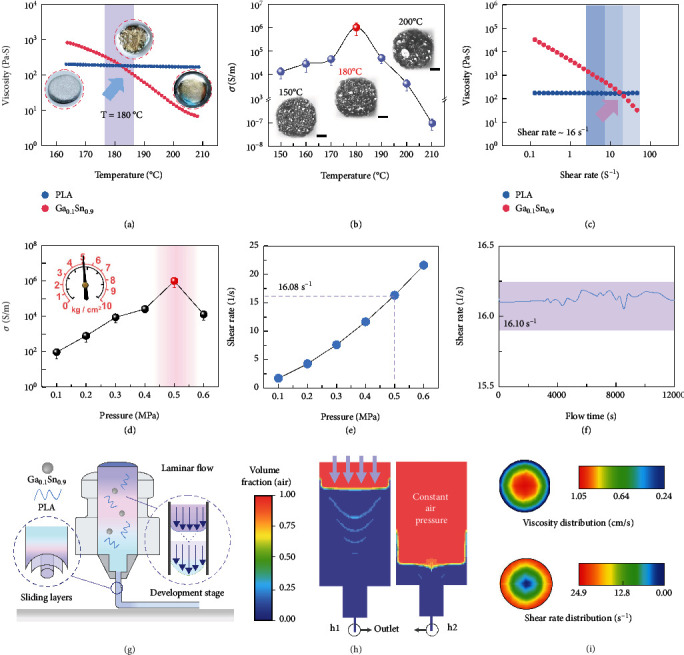
The processing conditions of SLMPFs. (a) Rheological curves for temperature sweep of PLA and Ga_0.1_Sn_0.9_ from 160 to 210°C. Two curves have an intersection at 180°C. Insets are the optical photos showing the morphology of Ga_0.1_Sn_0.9_ at 160°C, 180°C, and 210°C. (b) The volume conductivity (*σ*) of 55%-SLMPFs prepared under different processing temperatures. Insets are the cross-sectional morphology of 55%-SLMPFs prepared under 150°C, 180°C, and 200°C (scale bar:100 *μ*m). (c) Rheological curves for shear rate sweep of PLA and Ga_0.1_Sn_0.9_ from 0.1 to 100 s^−1^. Two curves have an intersection at 16 s^−1^. Blue shadow marks the shear rate range of the traditional plastic processing method. (d) The volume conductivity (*σ*) of 55%-SLMPFs changes with the processing pressure from 0.1 MPa to 0.6 MPa. Inset is a barometer of the injector showing the 5 kg/cm^3^ (0.5 MPa) pressure during injection. The red region highlights the optimal pressure in this work. (e) Numerical simulation gives the relationship between pressure and outlet shear rate. When the pressure is 0.5 MPa, the numerical simulation result of the average shear rate at the outlet is 16.08 s^−1^. (f) The shear rate under 0.5 MPa changes with the flow time output by numerical simulation software. The shear rate stabilizes around 16.10 s^−1^. (g) The cross-sectional illustration of injection setup utilized in this work to produce SLMPFs. Insets are illustrations of the fluid flow state inside the injector, which show the sliding layers and laminar flow progression stage of SLM polymer composites fluid. Fluid layers are independent with each other, resulting in relative movement. (h) Numerical simulation results of the distribution of air and SLM polymer composites during the injection. The red region represents air while the blue region represents SLM polymer composites. The blue waves show the trace of air in the SLM polymer composites. The grey circles the outlet. The details are shown in Movie S2. (i) The simulation of shear rate and velocity distribution at the outlet under the pressure of 0.5 MPa. Different colors represent the different numerical values.

Figure 3(a) shows the rheological curves of Ga_0.1_Sn_0.9_ and PLA from 160 to 210°C. Visually, insets in Figure 3(a) record the morphologies of Ga_0.1_Sn_0.9_ at 160°C, 180°C, and 210°C, which can be concluded that the phase states of SLM are significantly influenced by temperature. The temperature-dependent phase state strongly affects the rheological property of Ga_0.1_Sn_0.9_ under different temperatures [45].

The viscosity of Ga_0.1_Sn_0.9_ gradually decreases from 1000 to 5 Pa·S while that of PLA always stabilizes around 180 Pa·S. These two rheological curves have an intersection at 180°C, which means that the viscosity matching between SLM and PLA.  Figure 3(b) exhibits the volume conductivity of SLMPFs fabricated at various temperatures from 150 to 210°C, and the surfaces of the SLMPFs are shown in Figure S16. The SLMPFs fabricated at 180°C reach the highest conductivity (over 10^6^ S/m), which is at least one order of magnitude higher than those of SLMPFs prepared at other temperatures. Interestingly, the optimal temperature is the viscosity matching temperature (180°C) in Figure 3(a), indicating the intimate connection between high conductivity of SLMPFs and viscosity matching. Moreover, the thermal melting of SLMPFs occurs under 210°C (Figure S17), leading to a conductivity below 10^−8^ S/m. Inserts in Figure 3(b) are the cross-sectional morphology of 55%-SLMPFs prepared from 150°C, 180°C, and 200°C, showing that the processing temperature has important impact on the distribution of SLM in the PLA matrix. As shown in Figure S18, SLMPFs prepared under different temperatures have the similar high modulus (~300 MPa) due to the utilization of the same PLA matrix. Although the tensile stress and modulus are lower than those of neat PLA (Figure S19, Figure S20, and Table S1), the mechanical performance of SLMPFs is sufficient to fulfill the daily demands and exceeds many previously reported conductive fibers [14, 15]. As shown in the inset of Figure 2(f), the SLMPF can lift over 1 kg object which is about 1100 times the weight of fiber. Furtherly, under the intersected temperature (180°C), shear rate sweep is carried out and plotted in Figure 3(c). As the shear rate changes from 0.1 to 100 s^−1^, the Ga_0.1_Sn_0.9_ behaves as a typical shear thinning non-Newtonian fluid with viscosity decreased from 10^5^ to 12 Pa·S. Meanwhile, the viscosity of PLA is nearly independent of the shear rate within this range. Two rheological curves show an intersection point around 16 s^−1^ which locates in the representative shear rate range of classic polymer processing methods (Figure S21). As discussed in Figure 3(d), SLMPFs can achieve the highest conductivity under 0.5 MPa. Interestingly, numerical simulation results (Figure 3(e)) reveal that when the injection pressure is at 0.5 MPa, the corresponding shear rate is around 16 s^−1^, which is consistent with the rheological results of the intersection at 16 s^−1^ during the shear rate sweep (Figure 3(c)). As a result, the rheological characterization, numerical simulation, and the electrical conductivity of the SLMPFs' results mutually indicate that the viscosity matching of metals and polymers during the processing is critical to obtain the best fibers. We further explored the shear rate during the whole course of injection.  Figure 3(f) shows that the shear rate stabilizes around 16 s^−1^ with the extended flow time by numerical simulation software, revealing that the melt injection is steady and continuous.

To better understand the rheological behaviors, the rheological numerical simulation is conducted on Fluent software. Here, we utilized rheological curve of 55%-SLMPF (Figure S22) to fit the Cross-Williamson model [46]. The mathematical expression of the Cross-Williamson model is as follows.

(1) $$\begin{array}{c} \frac{\eta }{\eta_{0}}={\left[1+\left(\lambda \dot{\gamma }\right)\right]}^{\left(n-1\right)}, \end{array}$$

where *η*_0_, *λ*, $\dot{\gamma }$, and *n* represent zero shear viscosity, relaxation time, shear rate, and non-Newtonian index, respectively. The mathematical model matches the rheological curve of 55%-SLMPFs, and the *η*_0_, *λ*, and *n* can be obtained by the fitted curve.  Figure 3(g) shows the cross-sectional morphology of the injector utilized in this work (Figure S23). By calculating the Reynolds coefficient (less than 2000), we find that the movement of Ga_0.1_Sn_0.9_-PLA fluid in the barrel is laminar flow (Figure 3(g)). Laminar flow as a well-ordered flow again indicates the excellent thermal processability of Ga_0.1_Sn_0.9_-PLA composites. The relative movement between layers of laminar fluid leads to the generation of shear rate [47, 48]. The detailed simulation results are displayed in Movie S2, which visually reveals the well-ordered SLM polymer composites flow motion and development stage [49] in the injector.  Figure 3(h1) shows the simulation of the air distribution in the injector. A constant air pressure (0.5 MPa, represented by blue arrows) was applied to the barrel for the SLMPFs injection. The air volume percentage changes from 1 to 0 with the color gradually varying from red to dark blue (color bar).  Figure 3(h2) exhibits the state at the final moment of stable injection. When the volume fraction of SLM polymer composites in the tube is less than 0.1, the air pressure creates internal chaos (Figure S24), which is consistent with the phenomenon that the SLMPF injected at the end has irregular shape and is nonuniform. As shown in Figure 3(i), numerical simulation reveals that the shear rate and the velocity distribution at the outlet (shown in Figure 3(h)) are annular along the radius under the repressure of 0.5 MPa, which is following the basic theory of fluid flow [50, 51]. The fluid velocity reaches the maximum (1.05 cm/s) in the center of the pipe while close to zero around the wall of the pipe. The shear rate of fluid at the pipe center is close to zero and maximum (24.9 s^−1^) at the pipe wall. We also successfully fabricated the highly electrically conductive fibers at 160°C and 0.6 MPa (Figure S25), showing that the universal validity of controlling processing conditions to achieve the high conductivity of SLMPFs.

### 2.4. The Applications of SLMPFs

**Figure 4:**
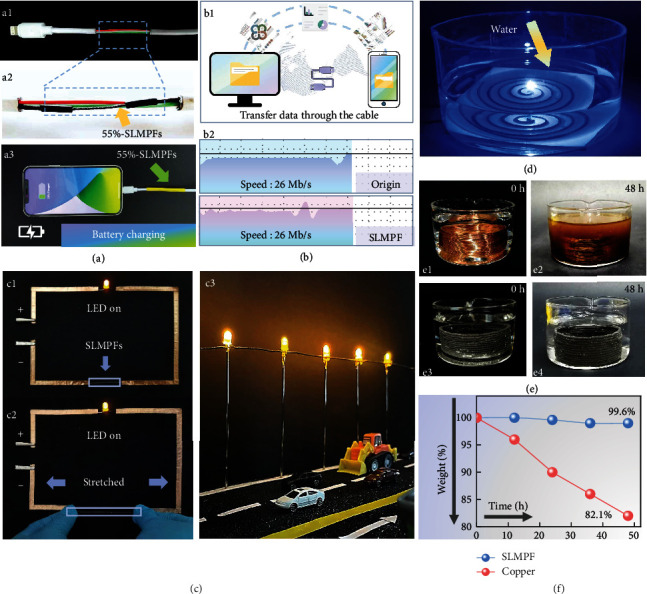
The applications of SLMPFs. (a1) A USB cable made by 55%-SLMPFs. A yellow arrow marks the 55%-SLMPF. (a2) Local enlarged image shows the junction details. (a3) A mobile phone was being charged by the SLMPF-made USB cable. (b1) Schematic illustration of data transmission between the computer and mobile phone. The transmission speeds through (b1) pristine USB cable and (b2) SLMPF-made USB cable. Both of them are around 26 Mb/s. (c) 55%-SLMPFs (TPU matrix) serve as the stretchable and electrical wires to light up LED (c1) before stretching and (c2) after stretching. (c3) Multiple LEDs are connected by a 55%-SLMPF. (d) The underwater LED was successfully lightened by 55%-SLMPFs circuit. (size: 10 cm × 10 cm, line diameter: 1.12 mm). (e) The comparison of (e1, e2) copper circuit and (e3, e4) SLMPFs' circuit after being electrified underwater for 48 h. (f) The weight change of SLMPFs' circuit and copper circuit being electrified underwater.

The 55%-SLMPFs can be melt-injected down to the minimum diameter of 200 *μ*m with a high filler loading, which is attributed to the excellent processability inherited from the polymer matrix (Figure S26). The 55%-SLMPFs (where is marked by the yellow arrow) replaced the copper wire in a commercial USB cable to configure the SLMPF-made USB cable, as shown in Figures 4(a1) and 4(a2). A mobile phone was stably charged via the SLMPF-made USB cable, and the charging process can rapidly respond to power without delay (Figure 4(a3) and Movie S3, the initial charge of the phone is 23%). Besides charging, the SLMPF-made USB cable also exhibits a functionality for data-transmitting. Figure 4(b) is the comparison of data transmission between the commercial USB cable and SLMPF-made USB cable, both transmission speed is around 26 Mb/s, showing that the performance of SLMPF-made USB cable is similar as the commercial products due to the superior conductivity of SLMPFs (Figure S27 and Movie S4). As shown in Figure 4(c), the LED was lighted up through the flexible and stretchable 55%-SLMPFs which uses TPU as polymer matrix, showing the potential application of SLMPFs as a stretchable circuit. The brightness of the LED connected by SLMPF (TPU matrix) has no obvious differences before (Figure 4(c1)) and after (Figure 4(c2)) stretching, indicating the electrical stability of SLMPFs under tensile force. Furthermore, SLMPFs also have the capability for underwater works because the polymer matrix can protect the fibers from water corrosion (Figure 4(d) and Movie S5). As shown in Figure 4(d), the immersed SLMPFs' circuit successfully lights the LED in the water. For evaluating the stability of the underwater SLMPFs' circuit, a 48 h continuous electrifying was applied to the SLMPFs circuit and copper circuit (Figure 4(e)). It is obvious that the copper circuit has been severely corroded (Figure 4(e2)) while SLMPFs' circuit has no change visually (Figure 4(e4)). Further, Figure 4(f) shows the SLMPFs' circuit has negligible weight change (0.4%) while the identical copper circuit loses the 17.9% weight due to the hydro-oxidization of copper (Figure S28 and Figure S29). With the outstanding electrical and waterproof performance, the SLMPFs could be utilized for a waterproof electrical interconnector in varied conditions.

## 3. Conclusion

In summary, this work successfully fabricated SLMPFs by the combination of semiliquid metal (SLM, gallium-tin alloy) and polymer matrix (PLA or TPU). The SLMPFs possess phenomenal electrical conductivity (over 10^6^ S/m), considerable tensile property (modulus: ~300 MPa), and excellent thermal processibility. The similar viscosity between SLMs and polymers during the melt-processing is the key to eliminate the phase segregation for such extraordinary electrical and mechanical performance. The rheological experiments and numerical simulation are extensively applied for the materials, which firmly supported the rheological matching principle. The super-conductive SLMPFs are suitable for scalable production by plastic molding, which can be broadly applied as electronics products, data communication, and underwater electrical instruments.

## 4. Materials and Methods

### 4.1. Materials

All materials used in this work are commercially available. Gallium (Ga, melting point at 29.8°C), tin (Sn, melting point at 232°C), copper (Cu, melting point at 1083°C), and iron (Fe, melting point at 1538°C) were purchased from Shenyang Jiabei Commercial Trading Company. Polylactic acid (PLA, NatureWorks Ingeo 3260HP, M_w_ = 10800, PDI = 1.61) and thermoplastic polyurethane (TPU, BASF SE 1190A, M_w_ = 10000) were purchased from Dongguan Qisuyu Plastics Materials Co., Ltd.

### 4.2. Preparation of Semiliquid Metal (SLM)

The Ga and Sn (atomic ratio of 1 : 9) were put into a 50 mL ceramic crucible and heated to 250°C for completely melting. Then, the Ga_0.1_Sn_0.9_ semiliquid metal (SLM) particles are formed by vigorously stirring during cooling under an ambient environment. Finally, the SLM powder (less than 100 *μ*m) was obtained by grinding and passing through a 100-mesh sieve.

### 4.3. Preparation of Semiliquid Metal Polymer Composite Fibers (SLMPFs)

Different volume fractions of SLM were mixed with polylactic acid (PLA) or thermoplastic polyurethane (TPU) in a 50 mL ceramic crucible and heated at 180°C to melt (in this paper, 55% of the volume fraction is selected). Then, the bulky SLM-PLA composites were moved into an injection mold and injected at 180°C. The resulted SLMPFs with regular diameters were obtained.

## Data Availability

All relevant data that support the findings are available within this article and Supplementary Materials.

## Supplementary Materials

Supplementary Materials Figure S1: The detailed fabrication process of the SLM polymer composites. Figure S2: The photos of different shapes of the injected 55%-SLMPFs. Figure S3: Cross-sectional SEM and EDS spectrum of a 55%-SLMPF. Figure S4: EDS mapping of gallium and tin discloses that the liquid region and solid region are mainly made of gallium and tin-gallium. Figure S5: The cross-sectional SEM of 55%-SLMPF (left) and 60%-SLMPF (right). Figure S6: The cross-sectional microscope images of a single 55%-SLMPF (TPU matrix) from different heights (scale bar: 100 *μ*m). Figure S7: The diagram of viscosity matching between SLM and PLA which is different from the phase separation between copper and polymer. Figure S8: DSC curves for SLM (Ga_0.1_Sn_0.9_), PLA, and SLMPFs at 2 °C/min. Figure S9: Cyclic DSC curves of the SLM (Ga_0.1_Sn_0.9_). Figure S10: The photos record the bottom morphology of Ga_0.1_Sn_0.9_ heated at 180 °C with (left) 0 h and (right) 6 h. Figure S11: The photos of a 55%-SLMPF which was heated at 180 °C for 30 minutes. Figure S12: Photos of different metal fillers under room temperature. Figure S13: The morphologies of (a) Fe-PLA, (b) Cu-PLA, and (c) Sn-PLA. Figure S14: The photo for Ga-PLA mixing. Figure S15: Cross-section and surface microscope of different SLMs (Ga_0.05_Sn_0.95_ and Ga_0.5_Sn_0.5_) as conductive fillers in the polymer matrix (The volume fraction of fillers is 55%). Figure S16: Photo of 55%-SLMPFs under different temperatures. Figure S17: Surface morphologies of 55%-SLMPFs injected from different temperatures under a certain processing pressure of 0.5 MPa. Figure S18: Tensile tests of 55%-SLMPFs prepared from different temperatures. Figure S19: The tensile curves for SLMPFs injected from different temperatures. Figure S20: The tensile curves for neat PLA. The PLA was injected from 180 °C and 0.5 MPa. Table S1: Detailed information of axial tensile tests for neat PLA and 55%-SLMPFs under different temperatures. Figure S21:. The schematic diagram of the shear rate of the classical polymer processing process. Figure S22: The rheological curve of 55%-SLMPFs and the fitting curve of the Cross- Williamson formula. Figure S23:.The 2D and 3D model of injection equipment we used in this work. Figure S24: The schematic diagram of internal chaos. Figure S25: The conductivity (*σ*) of 55%-SLMPFs prepared under different processing temperatures at 0.6 MPa. Figure S26:The morphologies of 55%-SLMPFs show the smallest diameters (182.8 *μ*m and 196.3 *μ*m) we achieved in this study. Figure S27: The photo of transmission speed between iPhone and personal computer by origin USB data line. Figure S28: Photos of the underwater circuit made from 55%-SLMPFs (left) and commercial copper (right). Figure S29: The weight change (%) of commercial copper and 55%-SLMPFs before and after power on. Movie S1: The electromagnetic induction of the coil wound by SLMPFs. Movie S2: The animation process of injection in this work is simulated by Fluent software. Movie S3: The mobile phone was charging by the 55%-SLMPF replaced USB data line. Movie S4: The transmission speed between iPhone and personal computer by 55%-SLMPFs USB data line. Movie S5: The LED bulb was lightened by a 55%-SLMPF spiral circuit under the water.

## References

[1] Dong K., Peng X., Wang Z. L. (2020). Fiber/fabric-based piezoelectric and triboelectric nanogenerators for flexible/stretchable and wearable electronics and artificial intelligence. *Advanced Materials*.

[2] Cui Z., He S.-A., Liu Q. (2020). Graphene-like carbon film wrapped tin (II) sulfide nanosheet arrays on porous carbon fibers with enhanced electrochemical kinetics as high-performance Li and Na ion battery anodes. *Advanced Science*.

[3] Wang H., Li R., Cao Y. (2022). Liquid metal fibers. *Advanced Fiber Materials*.

[4] Liu H., Xin Y., Lou Y., Peng Y., Wei L., Zhang J. (2020). Liquid metal gradient fibers with reversible thermal programmability. *Materials Horizons*.

[5] Yang L., Pan L., Xiang H., Fei X., Zhu M. (2022). Organic–inorganic hybrid conductive network to enhance the electrical conductivity of graphene-hybridized polymeric fibers. *Chemistry of Materials*.

[6] Park S., Baugh N., Shah H. K., Parekh D. P., Joshipura I. D., Dickey M. D. (2019). Ultrastretchable elastic shape memory fibers with electrical conductivity. *Advanced Science*.

[7] Yu Y., Guo J., Ma B., Zhang D., Zhao Y. (2020). Liquid metal-integrated ultra-elastic conductive microfibers from microfluidics for wearable electronics. *Science Bulletin*.

[8] Guo R., Wang H., Chen G., Yuan B., Zhang Y., Liu J. (2020). Smart semiliquid metal fibers with designed mechanical properties for room temperature stimulus response and liquid welding. *Applied Materials Today*.

[9] Chen G., Wang H., Guo R., Duan M., Zhang Y., Liu J. (2020). Superelastic EGaIn composite fibers sustaining 500% tensile strain with superior electrical conductivity for wearable electronics. *ACS Applied Materials & Interfaces*.

[10] Guo Y., Chen S., Sun L. (2021). Degradable and fully recyclable dynamic thermoset elastomer for 3D-printed wearable electronics. *Advanced Functional Materials*.

[11] Wang X.-X., Yu G.-F., Zhang J., Yu M., Ramakrishna S., Long Y. Z. (2021). Conductive polymer ultrafine fibers via electrospinning: preparation, physical properties and applications. *Progress in Materials Science*.

[12] Lai Y. C., Lu H. W., Wu H. M. (2021). Elastic multifunctional liquid–metal fibers for harvesting mechanical and electromagnetic energy and as self‐powered sensors. *Advanced Energy Materials*.

[13] Hiremath N., Mays J., Bhat G. (2017). Recent developments in carbon fibers and carbon nanotube-based fibers: a review. *Polymer Reviews*.

[14] Yu S., Wang X., Xiang H., Zhu L., Tebyetekerwa M., Zhu M. (2018). Superior piezoresistive strain sensing behaviors of carbon nanotubes in one- dimensional polymer fiber structure. *Carbon*.

[15] Yan T., Wang Z., Pan Z.-J. (2018). Flexible strain sensors fabricated using carbon-based nanomaterials: a review. *Current Opinion in Solid State and Materials Science*.

[16] Liu H., Huang Z., Chen T., Su X., Liu Y., Fu R. (2022). Construction of 3D MXene/silver nanowires aerogels reinforced polymer composites for extraordinary electromagnetic interference shielding and thermal conductivity. *Chemical Engineering Journal*.

[17] Liu H., Xin Y., Bisoyi H. K., Peng Y., Zhang J., Li Q. (2021). Stimuli-driven insulator-conductor transition in a flexible polymer composite enabled by biphasic liquid metal. *Advanced Materials*.

[18] Marinho B., Ghislandi M., Tkalya E., Koning C. E., de With G. (2012). Electrical conductivity of compacts of graphene, multi-wall carbon nanotubes, carbon black, and graphite powder. *Powder Technology*.

[19] Huang Y., Ellingford C., Bowen C., McNally T., Wu D., Wan C. (2020). Tailoring the electrical and thermal conductivity of multi-component and multi-phase polymer composites. *International Materials Reviews*.

[20] Wang H., Liu Z., Ding J. (2016). Downsized sheath–core conducting fibers for weavable superelastic wires, biosensors, supercapacitors, and strain sensors. *Advanced Materials*.

[21] Woo J., Lee H., Yi C. (2020). Ultrastretchable helical conductive fibers using percolated Ag nanoparticle networks encapsulated by elastic polymers with high durability in omnidirectional deformations for wearable electronics. *Advanced Functional Materials*.

[22] Chen S., Wang H.-Z., Zhao R.-Q., Rao W., Liu J. (2020). Liquid metal composites. *Matter*.

[23] Pan C., Markvicka E. J., Malakooti M. H. (2019). A liquid-metal-elastomer nanocomposite for stretchable dielectric materials. *Advanced Materials*.

[24] Haque F. M., Ishibashi J. S. A., Lidston C. A. L. (2022). Defining the macromolecules of tomorrow through synergistic sustainable polymer research. *Chemical Reviews*.

[25] Zhao Y., Wang X., Yang S. (2019). Protecting the nanoscale properties of ag nanowires with a solution-grown SnO2 monolayer as corrosion inhibitor. *Journal of the American Chemical Society*.

[26] Guo R., Yao S., Sun X., Liu J. (2019). Semi-liquid metal and adhesion-selection enabled rolling and transfer (SMART) printing: a general method towards fast fabrication of flexible electronics. *Science China Materials*.

[27] Peng Y., Liu H., Xin Y., Zhang J. (2021). Rheological conductor from liquid metal-polymer composites. *Matter*.

[28] Lv P., Yang X., Bisoyi H. K. (2021). Stimulus-driven liquid metal and liquid crystal network actuators for programmable soft robotics. *Materials Horizons*.

[29] Sivan V., Tang S.-Y., O'Mullane A. P. (2013). Liquid metal marbles. *Advanced Functional Materials*.

[30] Zhang W., Ou J. Z., Tang S.-Y. (2014). Liquid metal/metal oxide frameworks. *Advanced Functional Materials*.

[31] Zhao R., Guo R., Xu X., Liu J. (2020). A fast and cost-effective transfer printing of liquid metal inks for three-dimensional wiring in flexible electronics. *ACS Applied Materials & Interfaces*.

[32] Ren L., Xu X., Du Y., Kalantar-Zadeh K., Dou S. X. (2020). Liquid metals and their hybrids as stimulus-responsive smart materials. *Materials Today*.

[33] Daeneke T., Khoshmanesh K., Mahmood N. (2018). Liquid metals: fundamentals and applications in chemistry. *Chemical Society Reviews*.

[34] Sun X., Yuan B., Sheng L., Rao W., Liu J. (2020). Liquid metal enabled injectable biomedical technologies and applications. *Applied Materials Today*.

[35] Wang X., Lu C., Rao W. (2021). Liquid metal-based thermal interface materials with a high thermal conductivity for electronic cooling and bioheat-transfer applications. *Applied Thermal Engineering*.

[36] Guan Z., Wang L., Bae J. (2022). Advances in 4D printing of liquid crystalline elastomers: materials, techniques, and applications. *Materials Horizons*.

[37] Bueche F. (1972). Electrical resistivity of conducting particles in an insulating matrix. *Journal of Applied Physics*.

[38] Shang Y., He X., Li Y. (2012). Super-stretchable spring-like carbon nanotube ropes. *Advanced Materials*.

[39] Foroughi J., Spinks G. M., Aziz S. (2016). Knitted carbon-nanotube-sheath/spandex-core elastomeric yarns for artificial muscles and strain sensing. *ACS Nano*.

[40] Liu X., Tang C., Du X. (2017). A highly sensitive graphene woven fabric strain sensor for wearable wireless musical instruments. *Materials Horizons*.

[41] Cheng Y., Wang R., Sun J., Gao L. (2015). A stretchable and highly sensitive graphene-based fiber for sensing tensile strain, bending, and torsion. *Advanced Materials*.

[42] Granero A. J., Wagner P., Wagner K., Razal J. M., Wallace G. G., in het Panhuis M. (2011). Highly stretchable conducting SIBS-P3HT fibers. *Advanced Functional Materials*.

[43] Seyedin M. Z., Razal J. M., Innis P. C., Wallace G. G. (2014). Strain-responsive polyurethane/PEDOT:PSS elastomeric composite fibers with high electrical conductivity. *Advanced Functional Materials*.

[44] (2015). Hierarchically buckled sheath-core fibers for superelastic electronics, sensors, and muscles. *Science*.

[45] Flemings M. C., Riek R. G., Young K. P. (1976). Rheocasting. *Materials Science and Engineering*.

[46] Cross M. M. (1965). Rheology of non-Newtonian fluids: a new flow equation for pseudoplastic systems. *Journal of Colloid Science*.

[47] Munson B. R., Okiishi T. H., Rothmayer A. P., Huebsch W. W. (1990). Fundamentals of fluid mechanics. *Biofluid Mechanics*.

[48] Jordan A. M., Lee B., Kim K. (2019). Rheology of polymer multilayers: slip in shear, hardening in extension. *Journal of Rheology*.

[49] Ma Z., Zhang H., Fu H. (2022). Modelling flow-induced microstructural segregation in semi-solid metals. *Materials & Design*.

[50] Douglas J. F., Gasiorek J. M., Swaffield J. A. (1979). *Fluid Mechanics*.

[51] Astarita G., Marrucci G., Joseph D. D. (1975). Principles of non-Newtonian fluid mechanics. *Journal of Applied Mechanics*.

