# Supplementary material for: Scalable Fabrication of Metallic Conductive Fibers from Rheological Tunable Semi-Liquid Metals
Source: Research (Wash D C). 2022 Oct 27;2022:9890686. doi: 10.34133/2022/9890686 (PMC9639447; doi:10.34133/2022/9890686)
Supplement: Supplementary Materials — Figure S1: The detailed fabrication process of the SLM polymer composites. Figure S2: The photos of different shapes of the injected 55%-SLMPFs. Figure S3: Cross-sectional SEM and EDS spectrum of a 55%-SLMPF. Figure S4: EDS mapping of gallium and tin discloses that the liquid region and solid region are mainly made of gallium and tin-gallium. Figure S5: The cross-sectional SEM of 55%-SLMPF (left) and 60%-SLMPF (right). Figure S6: The cross-sectional microscope images of a single 55%-SLMPF (TPU matrix) from different heights (scale bar: 100 μm). Figure S7: The diagram of viscosity matching between SLM and PLA which is different from the phase separation between copper and polymer. Figure S8: DSC curves for SLM (Ga0.1Sn0.9), PLA, and SLMPFs at 2 °C/min. Figure S9: Cyclic DSC curves of the SLM (Ga0.1Sn0.9). Figure S10: The photos record the bottom morphology of Ga0.1Sn0.9 heated at 180 °C with (left) 0 h and (right) 6 h. Figure S11: The photos of a 55%-SLMPF which was heated at 180 °C for 30 minutes. Figure S12: Photos of different metal fillers under room temperature. Figure S13: The morphologies of (a) Fe-PLA, (b) Cu-PLA, and (c) Sn-PLA. Figure S14: The photo for Ga-PLA mixing. Figure S15: Cross-section and surface microscope of different SLMs (Ga0.05Sn0.95 and Ga0.5Sn0.5) as conductive fillers in the polymer matrix (The volume fraction of fillers is 55%). Figure S16: Photo of 55%-SLMPFs under different temperatures. Figure S17: Surface morphologies of 55%-SLMPFs injected from different temperatures under a certain processing pressure of 0.5 MPa. Figure S18: Tensile tests of 55%-SLMPFs prepared from different temperatures. Figure S19: The tensile curves for SLMPFs injected from different temperatures. Figure S20: The tensile curves for neat PLA. The PLA was injected from 180 °C and 0.5 MPa. Table S1: Detailed information of axial tensile tests for neat PLA and 55%-SLMPFs under different temperatures. Figure S21:. The schematic diagram of the shear rate of the classic [file 9890686.f1.zip › Proof Supplementary Materials.docx]

Supplementary Materials

Title

Scalable Fabrication of Metallic Conductive Fibers from Rheological Tunable Semi-Liquid Metals

**Authors**

Shujun Tian†, Hao Peng†, Huaizhi Liu, Jiancheng Zhou*, and Jiuyang Zhang*

**Affiliations**

School of Chemistry and Chemical Engineering, Jiangsu Hi-Tech Key Laboratory for Biomedical Research, Southeast University, Nanjing 211189, PR China.

Correspondence should be addressed to Jiancheng Zhou; jczhou@seu.edu.cn and Jiuyang Zhang; jiuyang@seu.edu.cn

†These authors contributed equally to this work.

**This file includes:**

Supplementary Text

Figures S1 to S29

Table S1

**Other Supplementary Materials for this manuscript include the following:**

Movies S1 to S5

**Supplementary Text**

**Experimental Procedures**

1. **Methods**

**1.1 Preparation of metal polymer composite fibers:** 55%Ga-PLA, 55%Sn-PLA, 55%Fe-PLA, and 55%Cu-PLA were prepared by the same method as the preparation of SLMPFs. All samples were melt-compounded at 180 ℃.

1. **Characterization**

**2.1 Injection molding:** The injection was carried out by loading the semi-liquid metal polymer composites into the injector (RZC-Z01, Origin Automation Technology Co., Ltd., Dongguan, China) and continuously injecting through a needle by exerting constant air pressure above with an air compressor BAIMING 600W-9L.

**2.2 Optical microscopy:** The microstructures of SLMPFs were observed in an optical microscope (XTL-16B).

**2.3 Differential scanning calorimeter (DSC):** The melting peak of Ga_0.1_Sn_0.9_, PLA, and SLM polymer composites were performed on DSC (TA DSC25) to characterize thermal transitions. Ga_0.1_Sn_0.9_, PLA, and SLM polymer composites were heated from 0 to 280 ℃ at a rate of 2 ℃/min.

**2.4** **Rheometer:** The rheological curves of Ga_0.1_Sn_0.9_, PLA, and SLM polymer composites were tested by rheometer (TA HR20) using a parallel plate geometry of 25 mm diameter. Before test, we mold the SLM to a cylinder (diameter: 25 mm; height: 1-2 mm). The rheological temperature sweep curves of PLA and Ga_0.1_Sn_0.9_ were measured from 160 to 210 ℃ with 5 ℃ /min at a shear rate of 16 s^-1^. The rheological shear rate sweep curves of PLA and Ga_0.1_Sn_0.9_ were measured from 0.1 to 100 s^-1^ under 180 ℃.

**2.5 Mechanical properties measurement:** The tensile test was performed on a tensile tester (MTS E42) at the ambient condition with a speed of 2 mm/min. All the reported values of mechanical properties were the average based on at least three independent measurements for each sample.

**2.6 Electrical properties measurement:** The electrical properties of the SLMPFs were measured by the multimeter and Keysight 34461A. The number of power line cycles (NPLC) and measurement range is 0.02 and automatic mode while the measurement option is Resistance 2 or 4 W. The wires linked the two or four sides of the cylindrical fiber to the Keysight 34461A. In the processes mentioned above, all the wires were fixed firmly by insulating tapes.

**2.7 Scanning electron microscope (SEM):** SEM images were observed by SSX-550, SHIMADZU Corporation with a voltage of 15 kV. The overlapped energy dispersive spectrometer (EDS) mapping was recorded using FEI Nova Nano SEM 450.

**2.8 Numerical simulation**

$\frac{\eta}{\eta_{0}}=\left[ 1+\left（ \lambda\dot{\gamma} \right） \right]^{\left（ n-1 \right）}$ (1)

$\eta_{0}$ is zero shear viscosity, ~6603.6 Pa·S. λ is relaxation time, ~6.3 s. n is the non-Newtonian index, 0.59 ± 0.1.

The theoretical model is established based on the Navier Stokes equation and continuity equation [1].
$\frac{\partial\left（ \rho u \right）}{\partial x}+\frac{\partial\left（ \rho v \right）}{\partial y}+\frac{\partial\left（ \rho w \right）}{\partial z}=0$ (2)

ρ is the fluid density, u, v and w represent the components of the velocity vector in the x, y, and z directions respectively.

Due to the complexity of material and motion changes in the extrusion process, in order to simplify the calculation process and save calculation resources, the following assumptions need to be made in the numerical calculation of the mixed extrusion process. (1) isothermal steady laminar flow, (2) no-slip wall, (3) incompressible melt, (4) no gravity and inertia, and (5) pure viscous fluid. Based on the above assumptions, the governing equation tensor form describing the flow field is as follows.

Momentum conservation equation: according to Newton's second law, the equations in x, y, and z directions are obtained.

- X-direction:
  $\nabla\cdot\left( \rho uV \right)=-\frac{\partial p}{\partial x}+\frac{\partial\tau_{xx}}{\partial x}+\frac{\partial\tau_{yx}}{\partial y}+\frac{\partial\tau_{zx}}{\partial z}$ (3)
- Y-direction:
   $\nabla\cdot\left( \rho vV \right)=-\frac{\partial p}{\partial y}+\frac{\partial\tau_{xy}}{\partial x}+\frac{\partial\tau_{yy}}{\partial y}+\frac{\partial\tau_{zy}}{\partial z}$ (4)
- Z-direction:
   $\nabla\cdot\left( \rho wV \right)=-\frac{\partial p}{\partial z}+\frac{\partial\tau_{xz}}{\partial x}+\frac{\partial\tau_{yz}}{\partial y}+\frac{\partial\tau_{zz}}{\partial z}$ (5)

ρ is the fluid density, p is pressure, V is the velocity vector, u, v and w represent the components of the velocity vector in the x, y, and z directions, $\tau_{xx}$, $\tau_{yx}$, $\tau_{zx} , \tau_{xy} , \tau_{yy},\tau_{zy} , \tau_{xz}, \tau_{yz}, \tau_{zz}$is a stress component in a rectangular coordinate system.

**2.9 Volume conductivity test:** The calculation formula of volume conductivity was concluded as follows:

$\sigma= \frac{L}{S\times R}$ (6)

L is the length of SLMPFs, m;

S is the cross-section area of SLMPFs, m^2^;

R is the resistance value of SLMPFs, Ω;

σ is the volume conductivity, S/m.


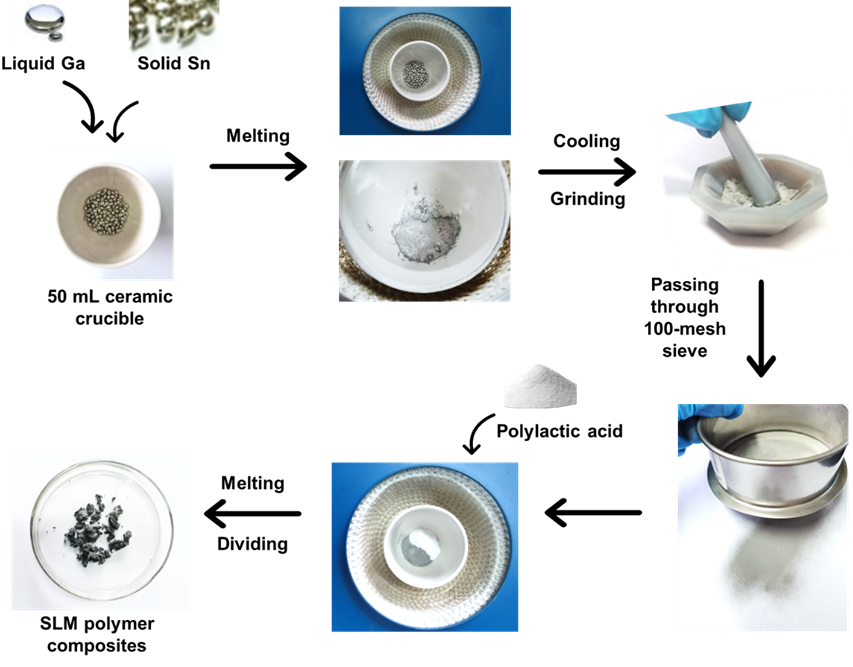


**Figure S1.** The detailed fabrication process of the SLM polymer composites. Metal Ga and Sn (atomic ratio of 1:9) were put into a 50 mL ceramic crucible and heated to melting. Then, the Ga-Sn was stirred and cooled at an ambient environment. Finally, the SLM powder (less than 100 µm) was obtained by grinding and passing through a 100-mesh sieve. Different volume fractions of SLM and PLA were put into a 50 mL ceramic crucible and heated at 180 ℃ to melt. After dividing it into small pieces, the mixture was moved into an injection mold and injected at 180 ℃.


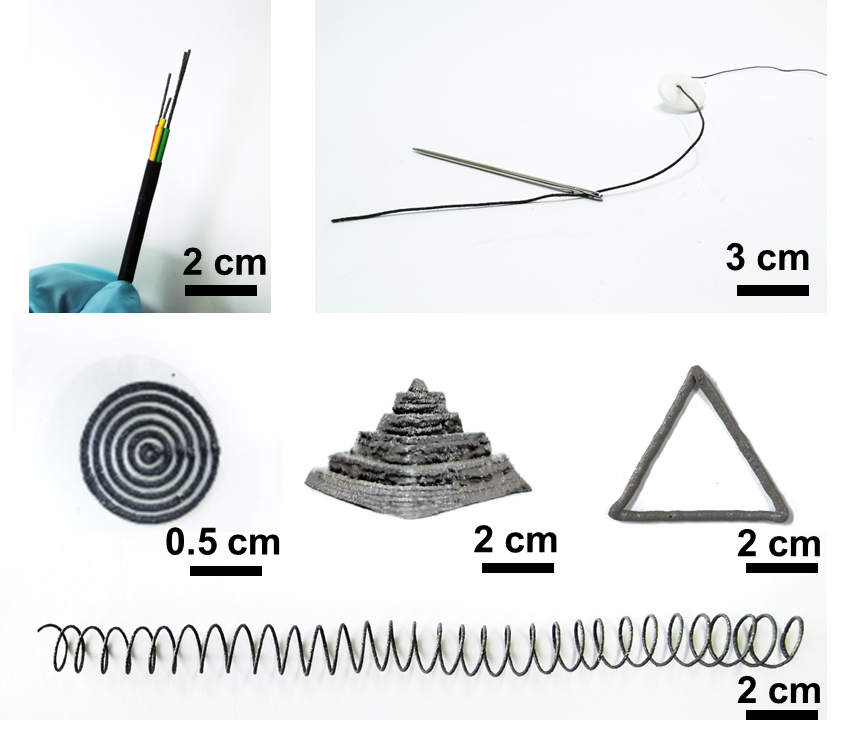


**Figure S2.** The photos of different shapes of the injected 55%-SLMPFs, such as cables, thread, concentric circles, pyramid, equilateral triangle, and spring. It indicates that the 55%-SLMPFs successfully inherit the excellent processability from PLA.


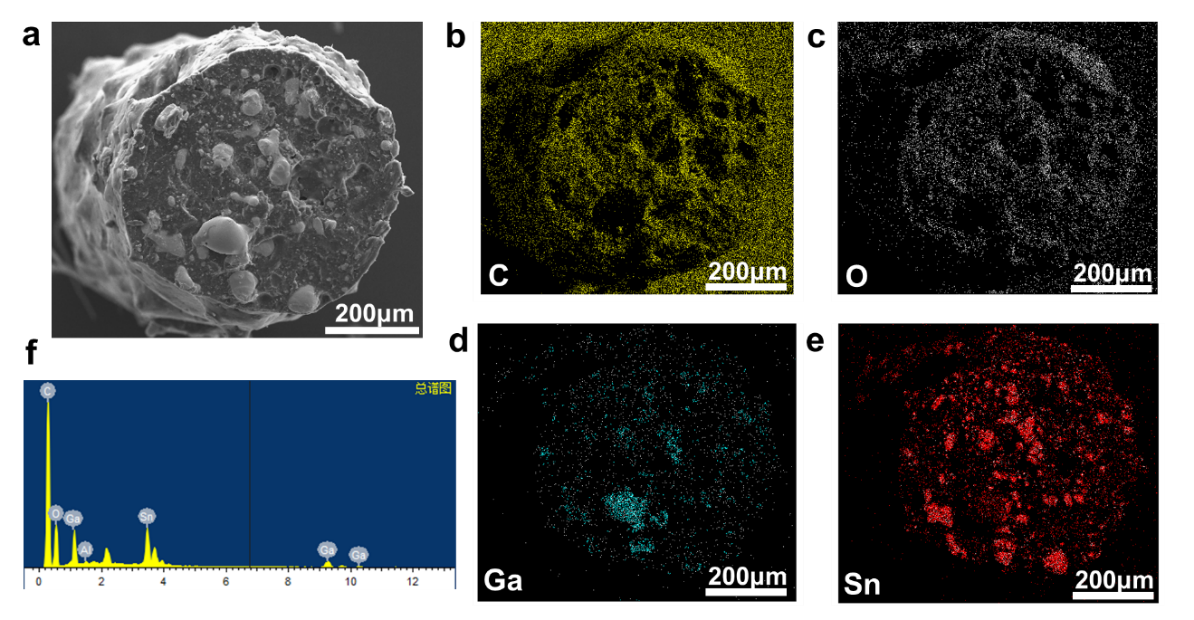


**Figure S3.** Cross-sectional SEM and EDS spectrum of a 55%-SLMPF. (a) Cross-sectional SEM of a 55%-SLMPF. (b-e) EDX elemental maps of b) carbon, d) oxygen, d) gallium, and e) tin of the sample are shown in (a). (f) EDS spectrum of the cross-section of the sample shown in (a). The quantity of each element conforms to the feeding proportion.

**
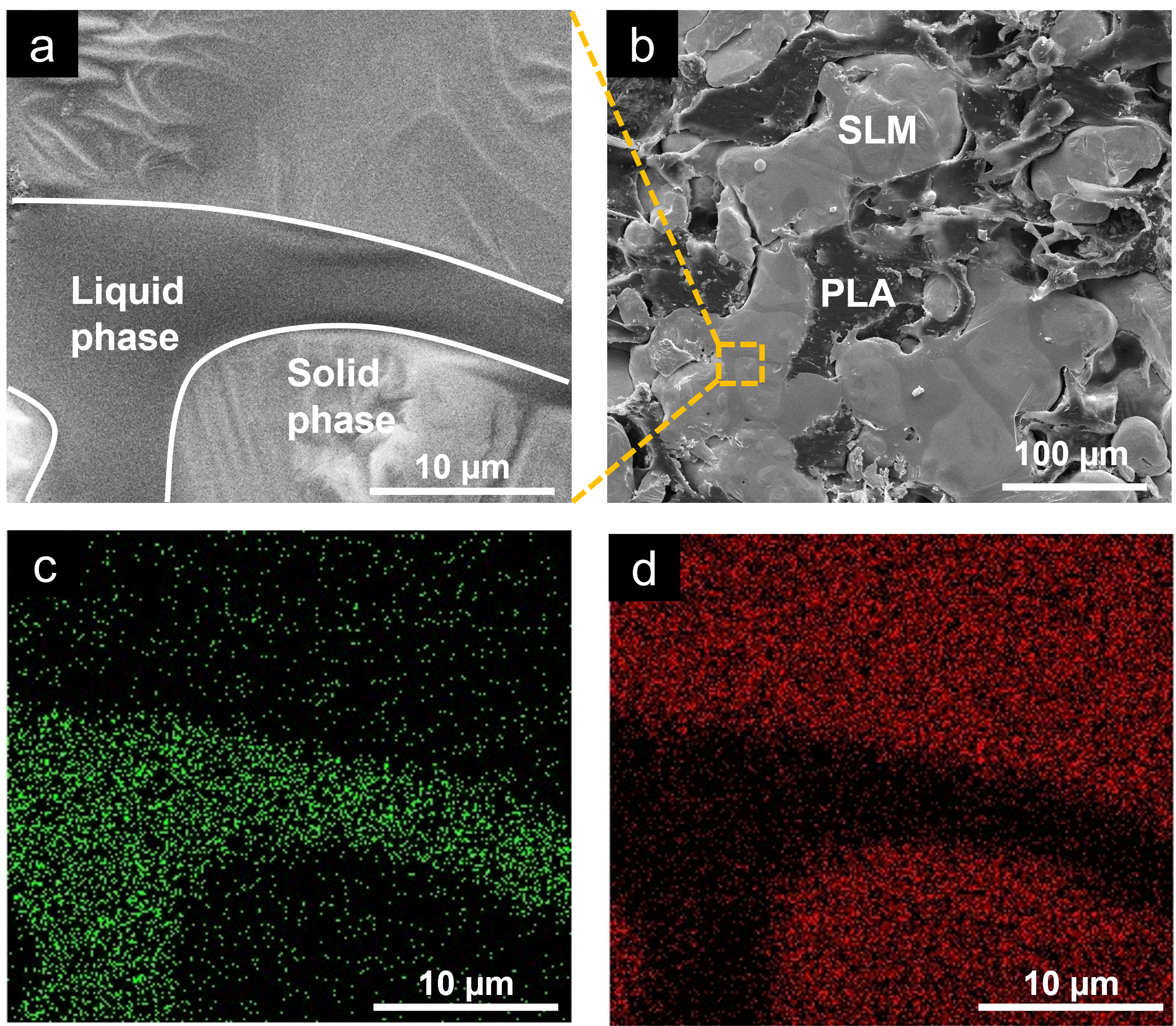
**

**Figure S4.** a) Local enlarged SEM image of b), the white lines mark the boundary between the solid phases and liquid phase. b) The cross-sectional SEM image of 55%-SLMPF. Dark and bright regions on the metal particles' surface represent the liquid phase and solid phase, respectively. EDS mapping of c) gallium and d) tin disclose that the liquid region and solid region are mainly made of gallium and tin-gallium, respectively, which consists with the gallium-tin phase diagram.


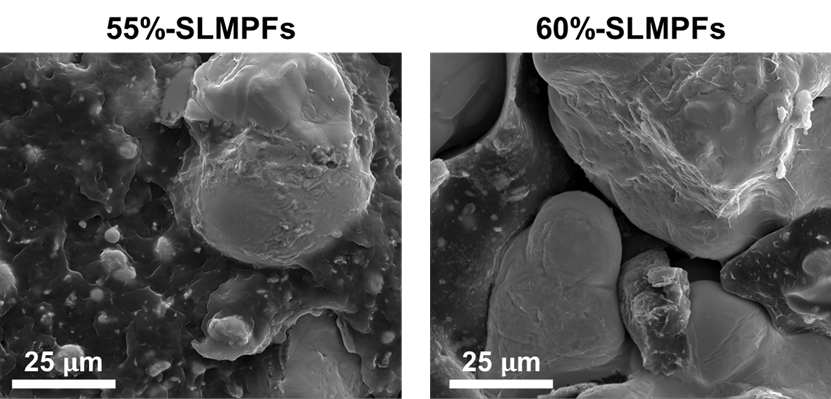


**Figure S5.** The cross-sectional SEM of 55%-SLMPF (left) and 60%-SLMPF (right). It can be seen that the 60% loading is too high to form a uniform distribution in the polymer matrix.


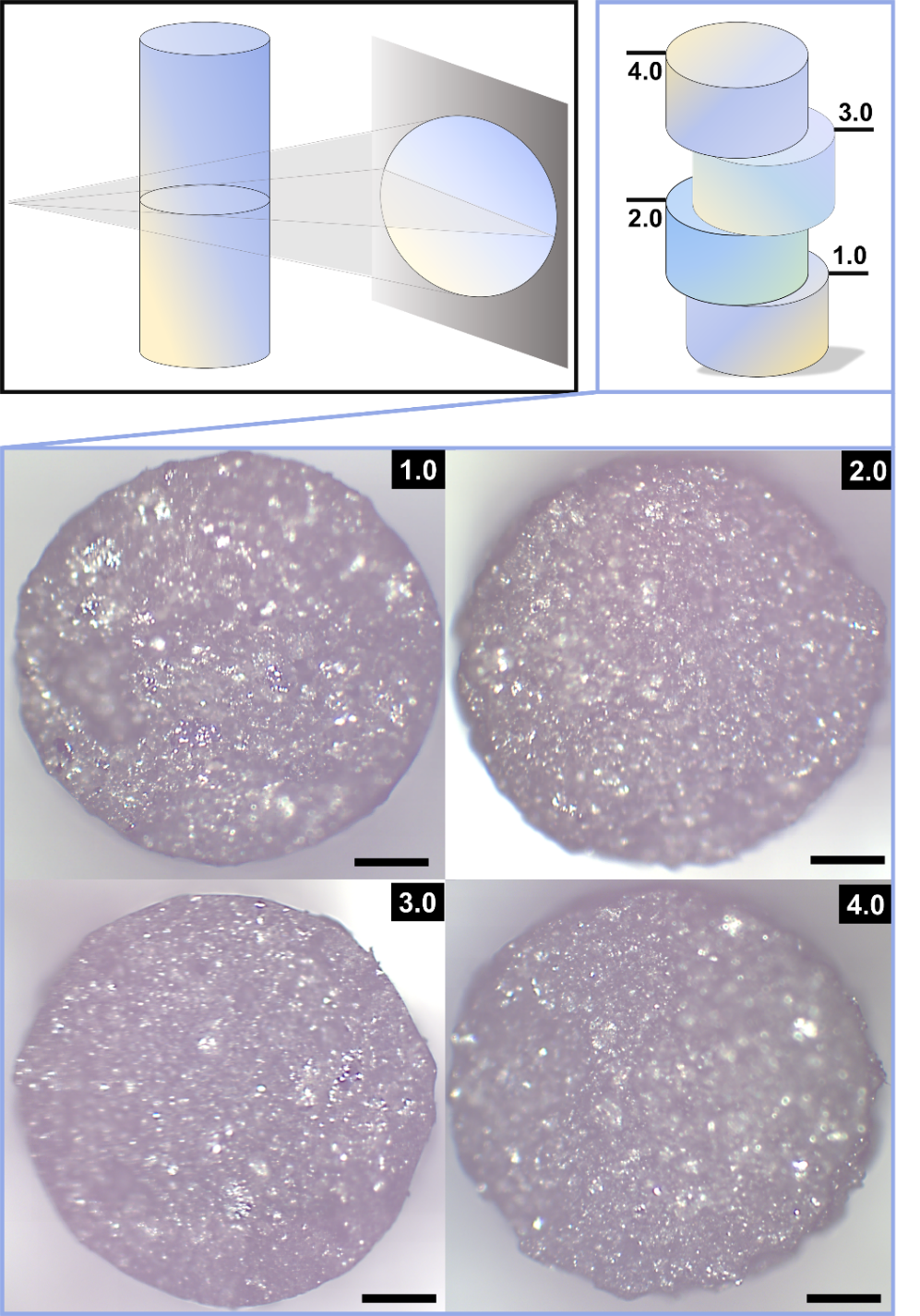


**Figure S6.** The cross-sectional microscope images of a single 55%-SLMPF (TPU matrix) from different heights (scale bar: 100 µm). The numbers in the right top corners denote the location (height from the bottom) where to be observed.


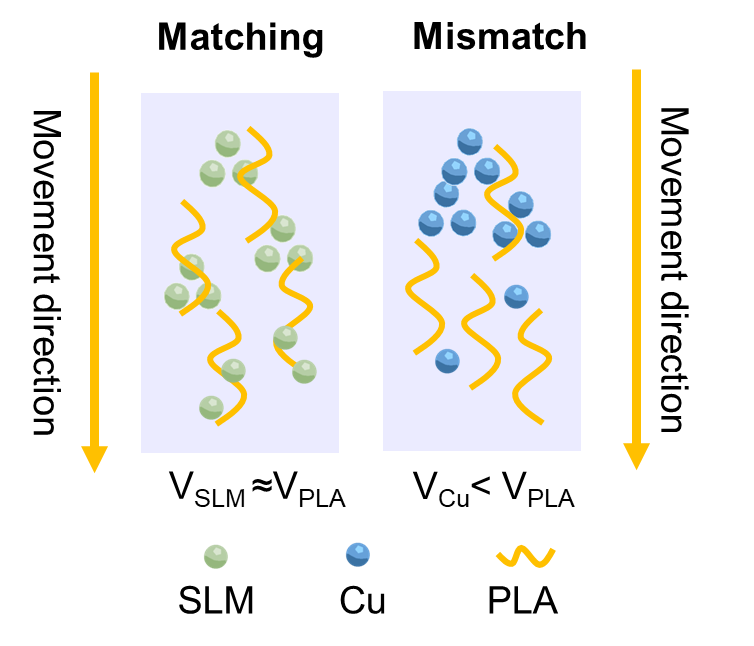


**Figure S7.**  The scheme describes the movement of SLM, Cu, and PLA in one certain direction during processing. The SLM and PLA have identical movement velocity (V_SLM_≈V_PLA_) under identical processing conditions due to the viscosity matching between SLM and PLA, leading to a uniform distribution. By comparison, the Cu moves much slower than PLA (V_Cu_≪V_PLA_) due to the giant viscosity difference, resulting in a phase segregation between Cu and PLA.

**Figure S8**. The melting peak of Ga_0.1_Sn_0.9_, PLA, and SLM polymer composites were performed on DSC (TA DSC 25) to characterize thermal transitions. Ga_0.1_Sn_0.9_, PLA, and SLM polymer composites were heated from 0 to 280 ℃ at a rate of 2 ℃/min. The melting peak of the DSC curve for PLA is 116.0 °C. The solidus temperature peak of the DSC curve for Ga_0.1_Sn_0.9_ is 20.5 °C and the liquidus temperature peak is 209.0 °C.

**Figure S9.** Cyclic DSC curves of the Ga_0.1_Sn_0.9_. Dotted lines mark the liquidus temperature and solidus temperature of Ga_0.1_Sn_0.9_, showing the stable composition of Ga_0.1_Sn_0.9_ under cyclic heating and cooling.


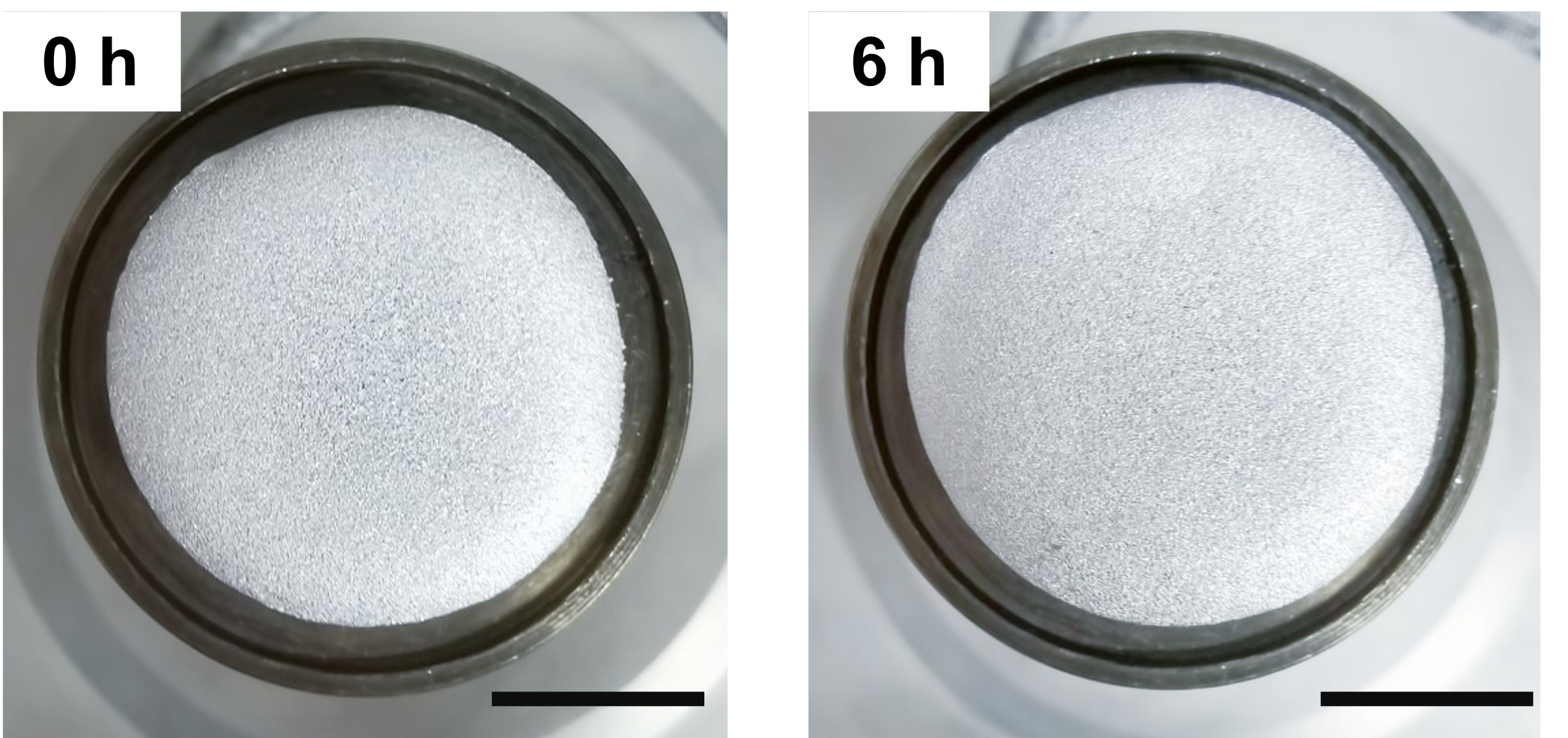


**Figure S10.** The photos record the bottom morphology of Ga_0.1_Sn_0.9_ heated at 180 °C with (left) 0 h and (right) 6 h. Scale bar: 1 cm. There is no different before and after rheology measurements, indicating the stable metal samples.

**
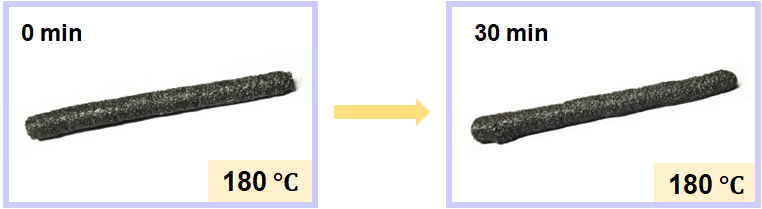
**

**Figure S11**. The photos of a 55%-SLMPF which was placed on the heater and heated at 180 ℃ for 30 minutes. It presents that there was no gallium leakage on the surface of fiber when the temperature rises.

**
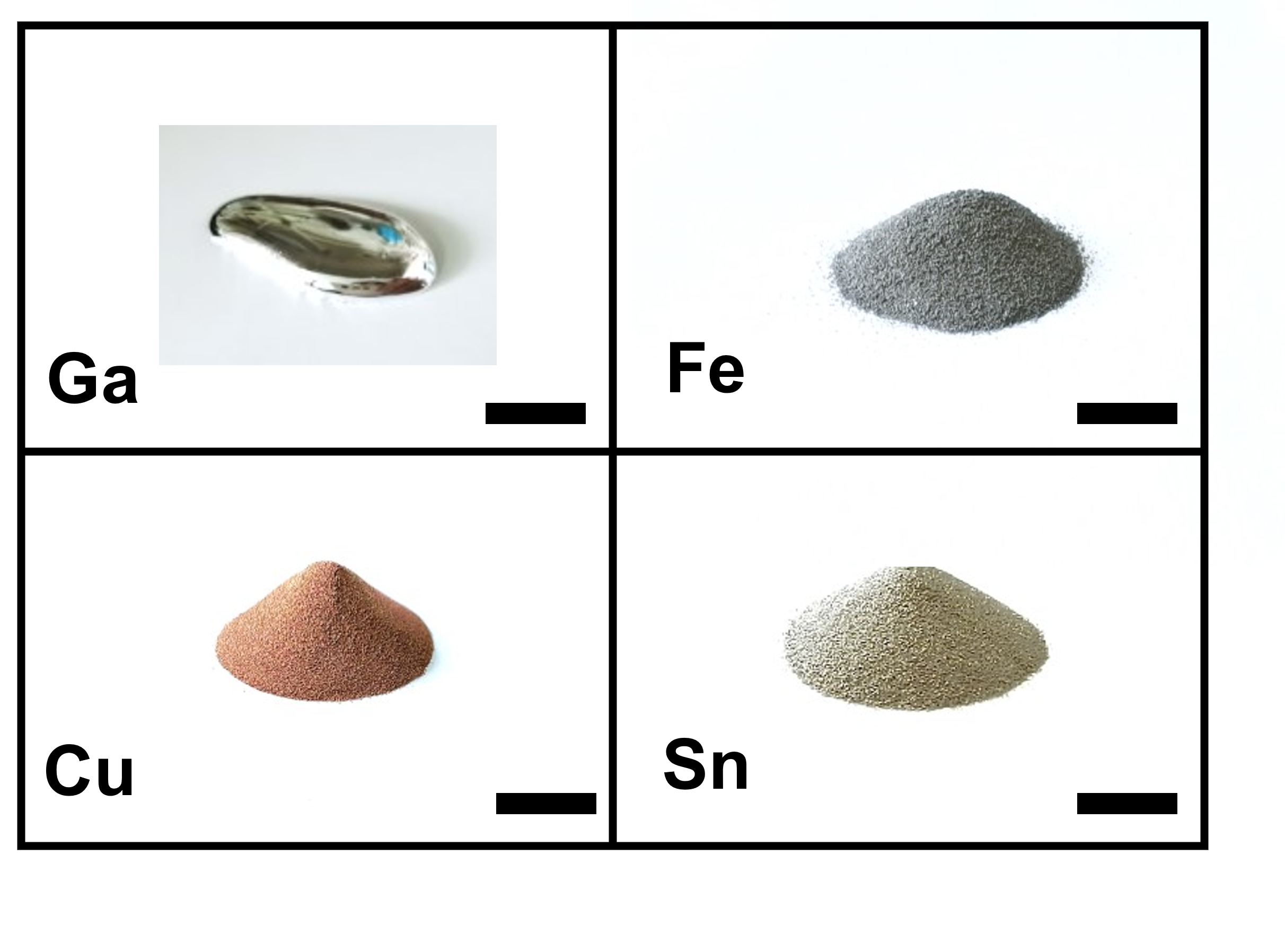
**

**Figure S12.** Photos of different metal elements under room temperature: gallium, iron, copper, and tin (scale bar: 1 cm).

**
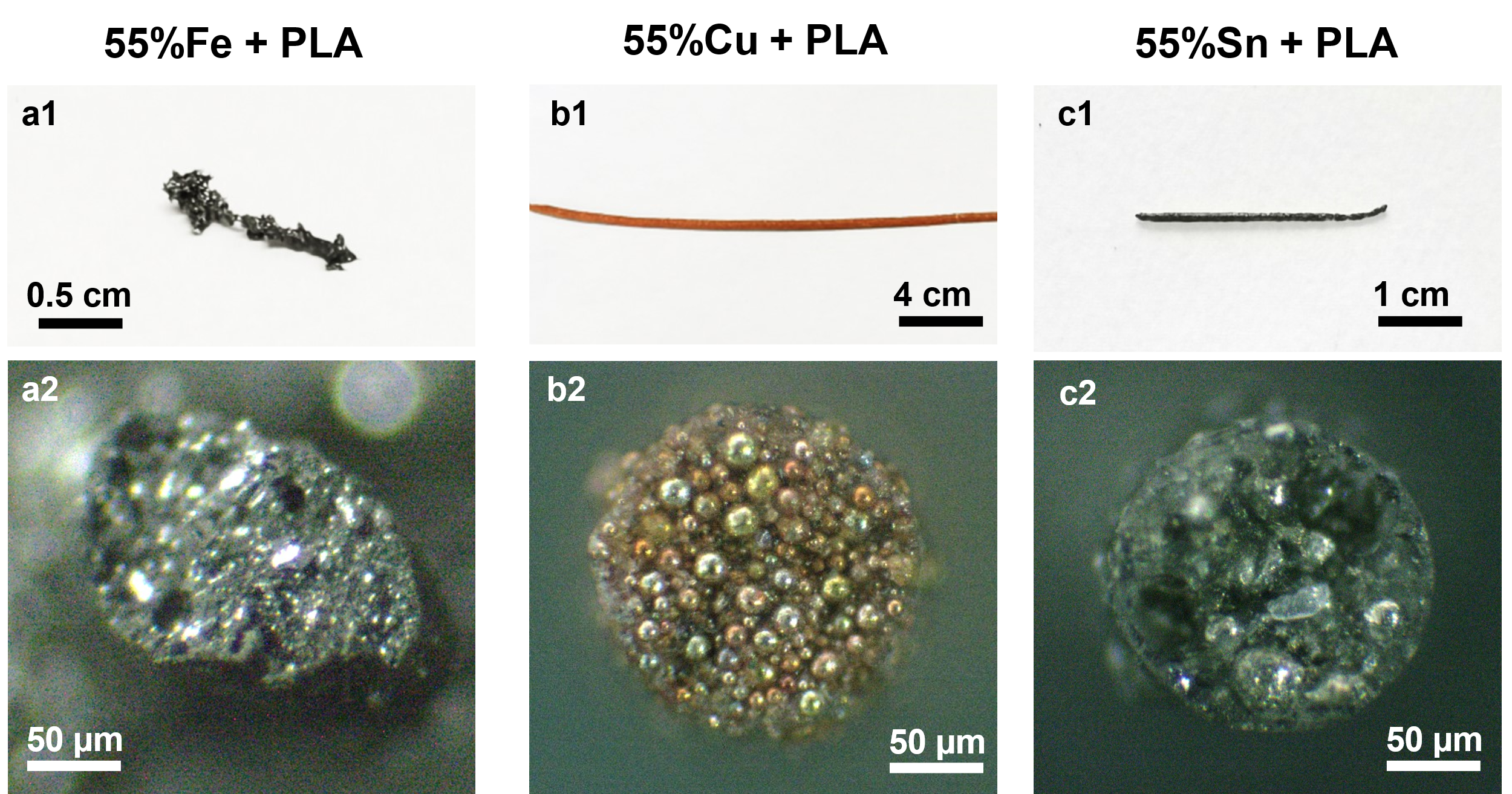
**

**Figure S13.** The morphologies of (a) Fe-PLA, (b) Cu-PLA, and (c) Sn-PLA. From the photos (a1, b1, and c1), compared with the SLMPFs (**Figure S3**), the hard metal particles (Fe, Cu, and Sn particles) damage the processability of composites because the metal particles are severely aggregated from microscopic images (a2-c2).


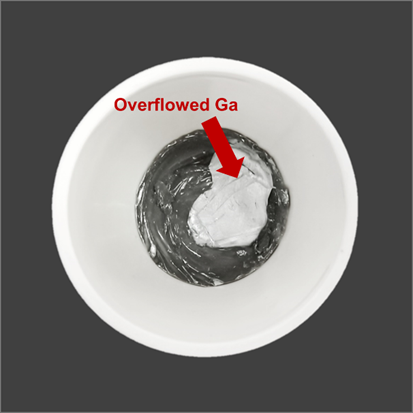


**Figure S14.** The photo recorded the mixing of Ga (55 vol%) and PLA. The mixing was conducted under 180 °C with a stirring at 50 rpm for 5 min. During the whole process, the Ga is immiscible with PLA all the time, attributing that the viscosity of PLA is nearly five magnitude orders higher than that of Ga (Matter, 2020, 2, 1446 -1480).

**
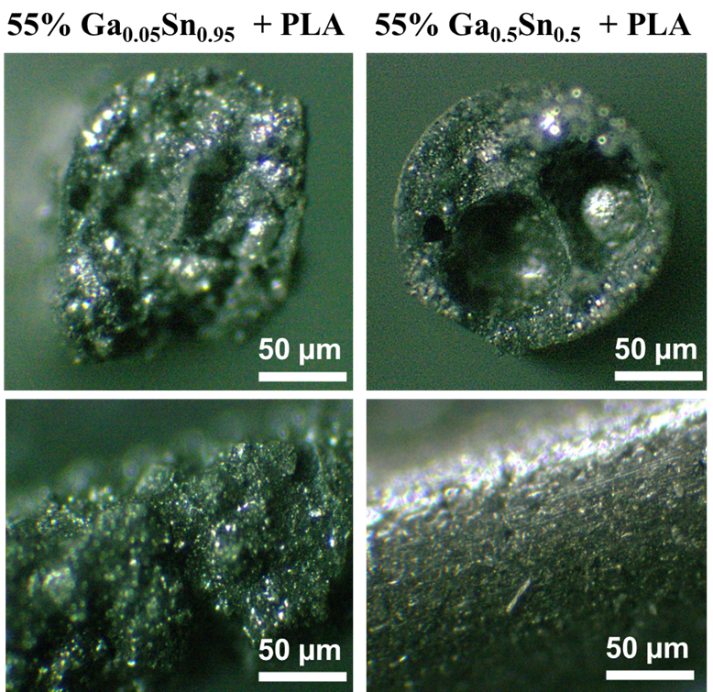
**

**Figure S15.** Cross-section and surface microscope of different SLMs (Ga_0.05_Sn_0.95_ and Ga_0.5_Sn_0.5_) as conductive fillers in the polymer matrix (The volume fraction of fillers is 55%). Compared with pure metals, SLMs have a unique solid-liquid state which leads to adjustable viscosity for matching with PLA under processing. Due to the different solid fractions of SLM, the conductivity of Ga_0.05_Sn_0.95_-PLA and Ga_0.5_Sn_0.5_-PLA were lower than Ga_0.1_Sn_0.9_-PLA (Identical loading: 55%). The Cross-sectional and surface morphologies of Ga_0.05_Sn_0.95_-PLA show that Ga_0.05_Sn_0.95_ in PLA is wrapped by polymers liking the pure metal in **Figure S13**. The Cross-sectional and surface morphologies of Ga_0.5_Sn_0.5_-PLA show that Ga_0.5_Sn_0.5_ in PLA severely agglomerates.


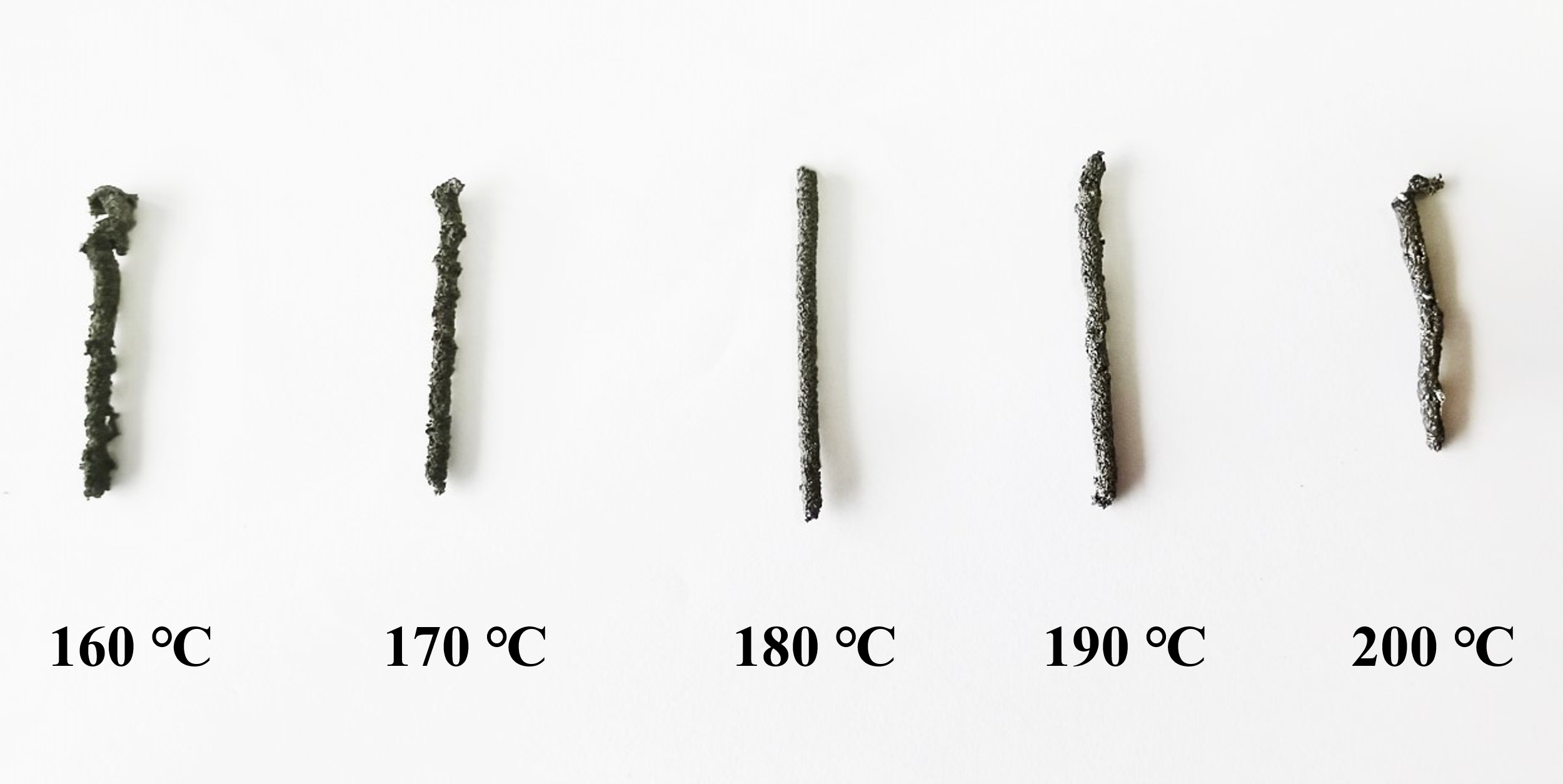


**Figure S16.** The photo of 55%-SLMPFs under different temperatures (160 ℃, 170 ℃, 180 ℃, 190 ℃, and 200 ℃). From the morphology, it can be seen that 180 ℃ is suitable for 55%-SLMPFs preparation.


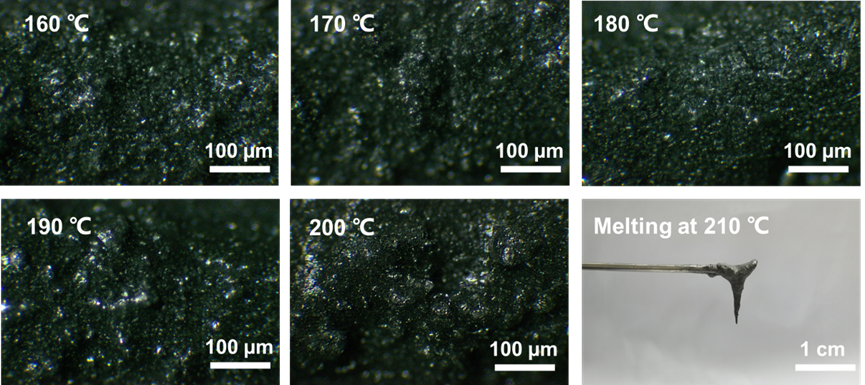


**Figure S17**. Surface morphologies of 55%-SLMPFs injected from different temperatures (160 ℃, 170 ℃, 180 ℃, 190 ℃, and 200 ℃) under a certain processing pressure of 0.5 MPa. Compared with others, the SLMPFs injected from 180 ℃ had the smoothest surface due to the viscosity matching between SLM and PLA. SLM polymer composites melted at 210 ℃.


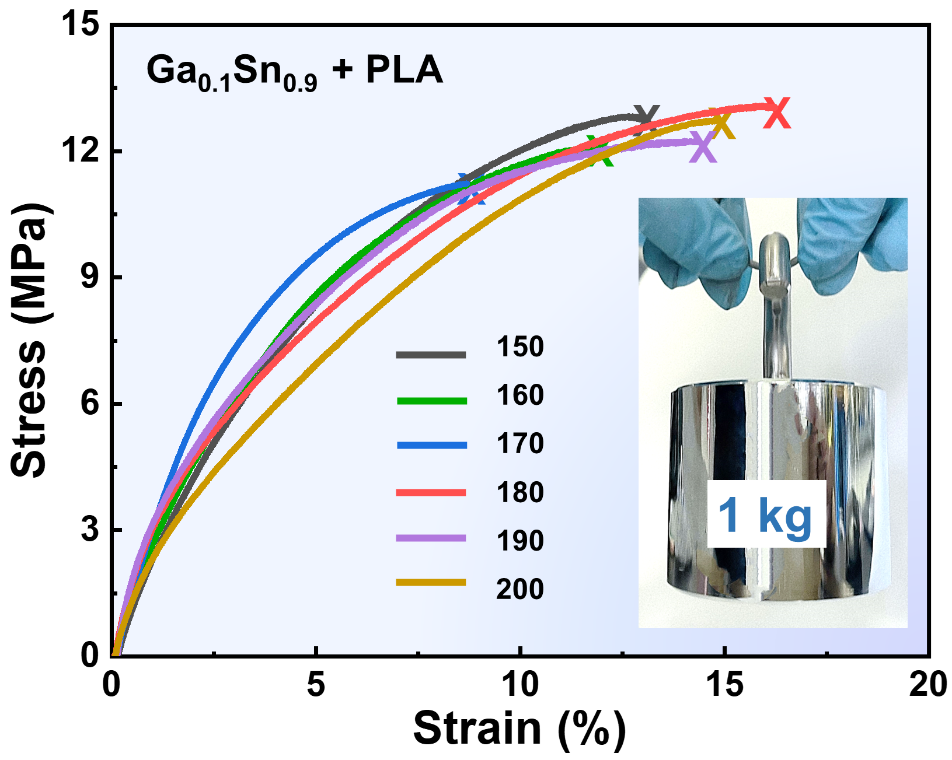


**Figure S18.** Tensile tests of 55%-SLMPFs prepared from different temperatures. Inset shows a single fiber (diameter: 1 mm) can carry a load of 1 kg which is about 1100 times the weight of fiber.


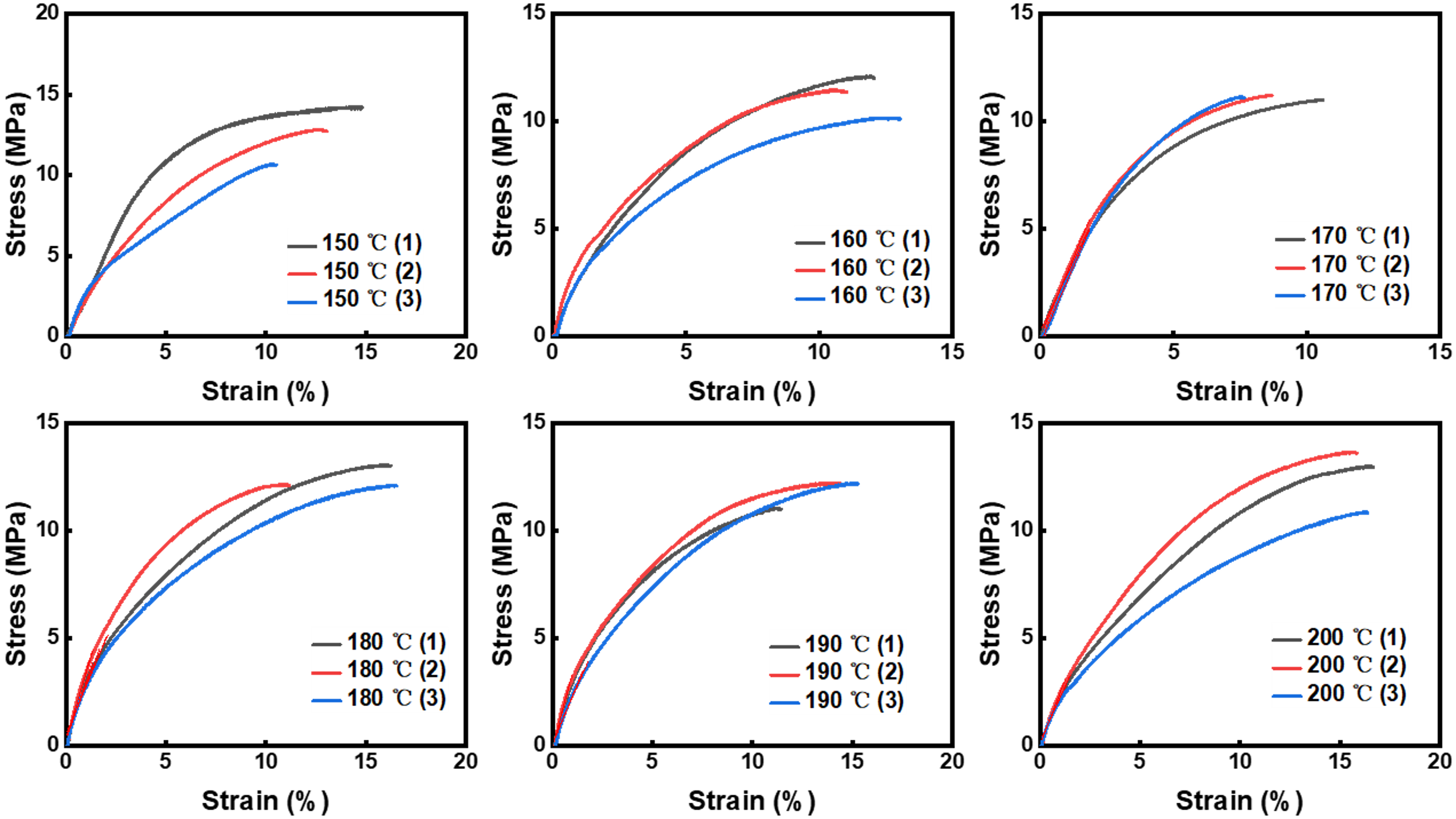


**Figure S19**. The tensile curves for SLMPFs injected from different temperatures (150 ℃, 160 ℃, 170 ℃, 180 ℃, 190 ℃, and 200 ℃). The tensile tests were performed on a tensile tester (MTS E42) at the ambient condition with a speed of 2 mm/min. Three identical samples were tested to evaluate the mechanical performance.

**Figure S20**. The tensile curves for neat PLA. The PLA was injected from 180 ℃ and 0.5 MPa. The tensile tests were performed on a tensile tester (MTS E42) at the ambient condition with a speed of 2 mm/min. Three identical samples were tested to evaluate the mechanical performance.

**Table S1.** Detailed information of axial tensile test for neat PLA and 55%-SLMPFs under different temperatures (150 °C, 160 °C, 170 °C, 180 °C, 190 °C, and 200 °C).

| **Samples** | **Elongation**  **at break**  **(*ε_b_*, %)** | **Tensile stress**  **at break**  **(*σ_b_*, MPa)** | **Young’s modulus**  **(E, MPa)** | **Toughness**  **(MJ/m^3^)** |
| --- | --- | --- | --- | --- |
| **Neat PLA** | 11.03 ± 1.68 | 31.75 ± 8.11 | 560 ± 87 | 2.25 ± 0.84 |
| **150** °C | 12.8 ± 2.3 | 12.5 ± 1.9 | 261 ± 37 | 1.15 ± 0.45 |
| **160** °C | 12.1 ± 1.0 | 11.3 ± 1.1 | 247 ± 21 | 0.96 ± 0.06 |
| **170** °C | 8.92 ± 1.6 | 11.11 ± 0.13 | 276 ± 27 | 0.7 ± 0.15 |
| **180** °C | 16.5 ± 3.51 | 12.4 ± 0.66 | 301 ± 30 | 1.31 ± 0.33 |
| **190** °C | 13.61 ± 2.34 | 11.8 ± 0.77 | 226 ± 16 | 1.16 ± 0.27 |
| **200** °C | 15.5 ± 1.13 | 12.39 ± 1.54 | 216 ± 25 | 1.39 ± 0.20 |


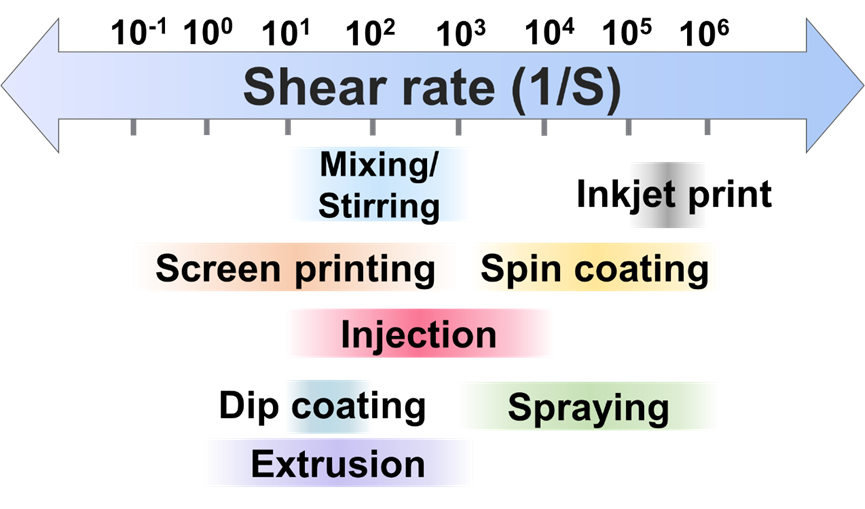


**Figure S21**. The schematic diagram of the shear rate of the classical polymer processing process [2, 3].


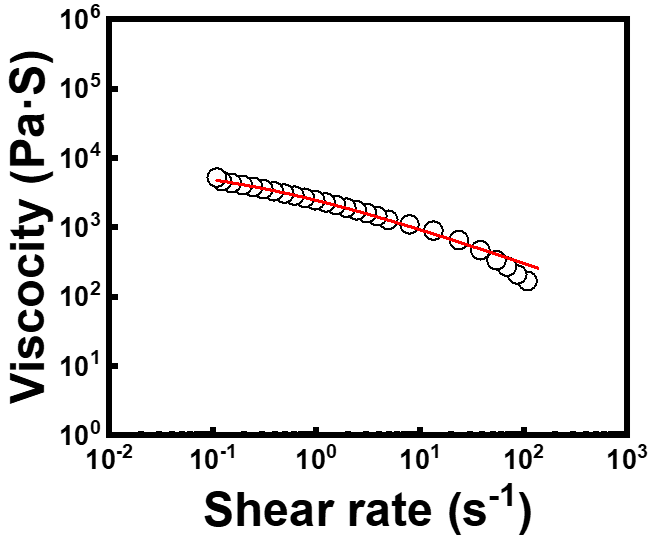


**Figure S22.** The rheological curve of 55%-SLMPFs and the fitting curve of the Cross Williamson formula. Rheological shear rate sweep curve of 55% -SLM polymer composites under 180 °C. The black circles represent the measured data and the red line represents the fitting curve of the Cross Williamson formula.


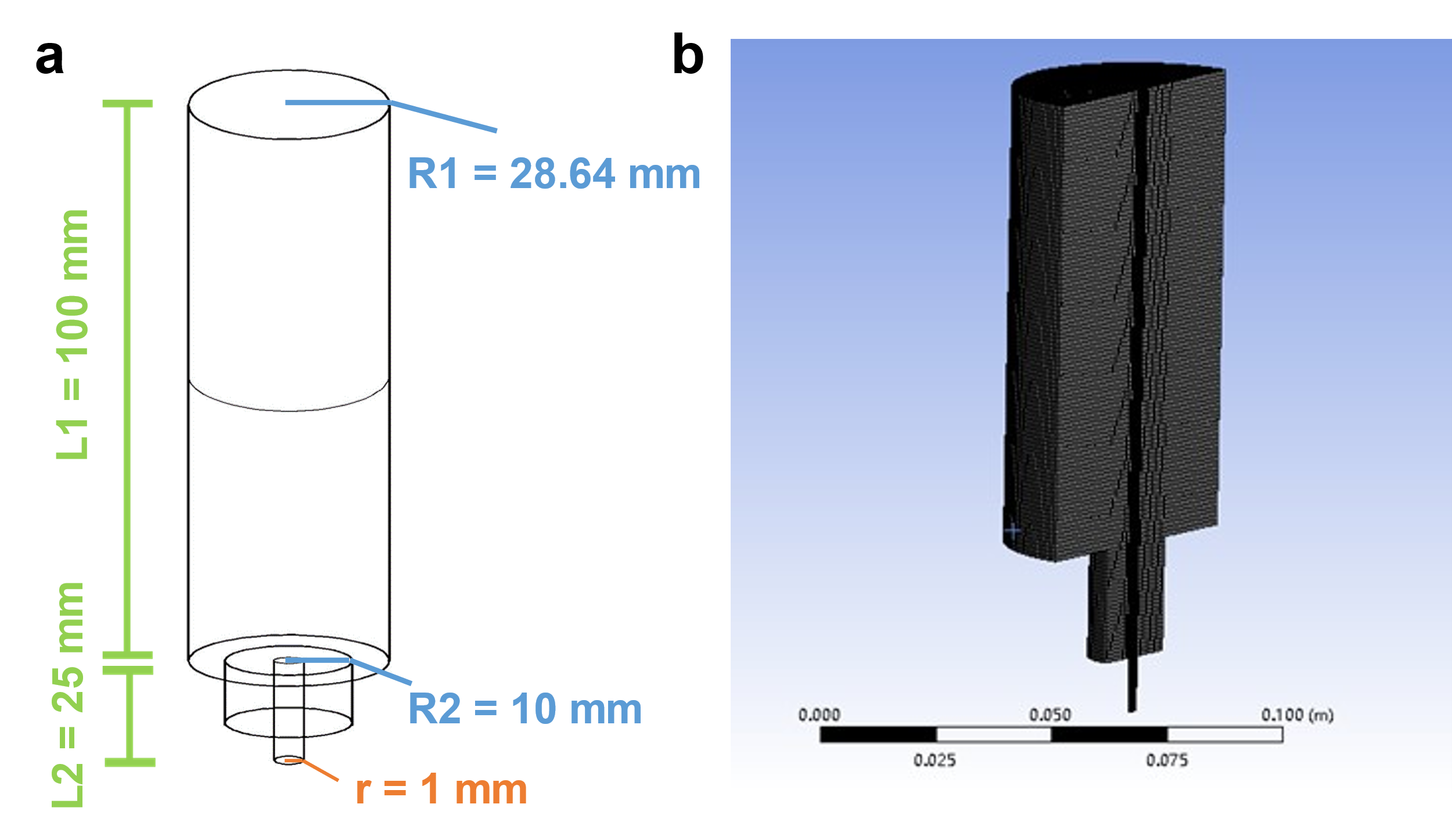


**Figure S23**. Modeling of the injector. (a) The 2D plan of injection equipment we used in this work. The accurate value of the injection machine including radius and length is shown in the diagram. (b) The section of the 3D injection model is built based on the CFD software. The radius and length are consistent with the data shown in (a).


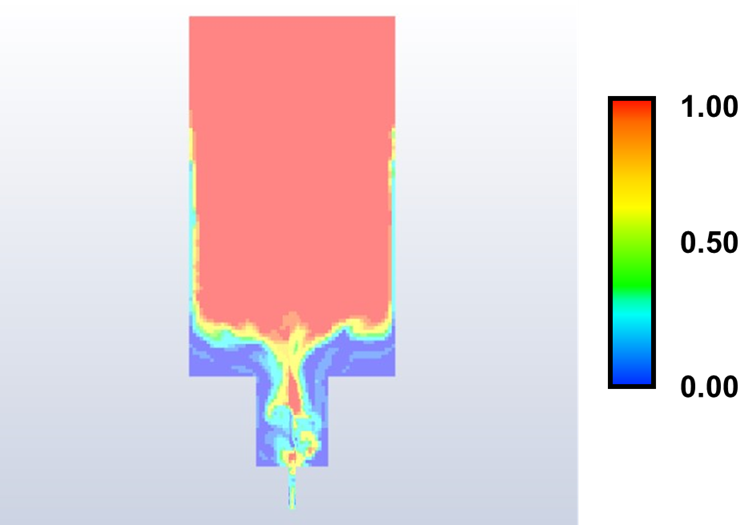


**Figure S24.** The schematic diagram of internal chaos when the volume of SLM polymer composites in the tube is less than that required for normal processing. The volume fraction of air in the red region is 1 and in the dark blue region is 0. However, the volume fraction of SLM polymer composites in the red region is 0 and in the dark blue region is 1. The intermediate transition color region represents that airflow penetrates the SLM polymer composites due to the complex injection process.

**
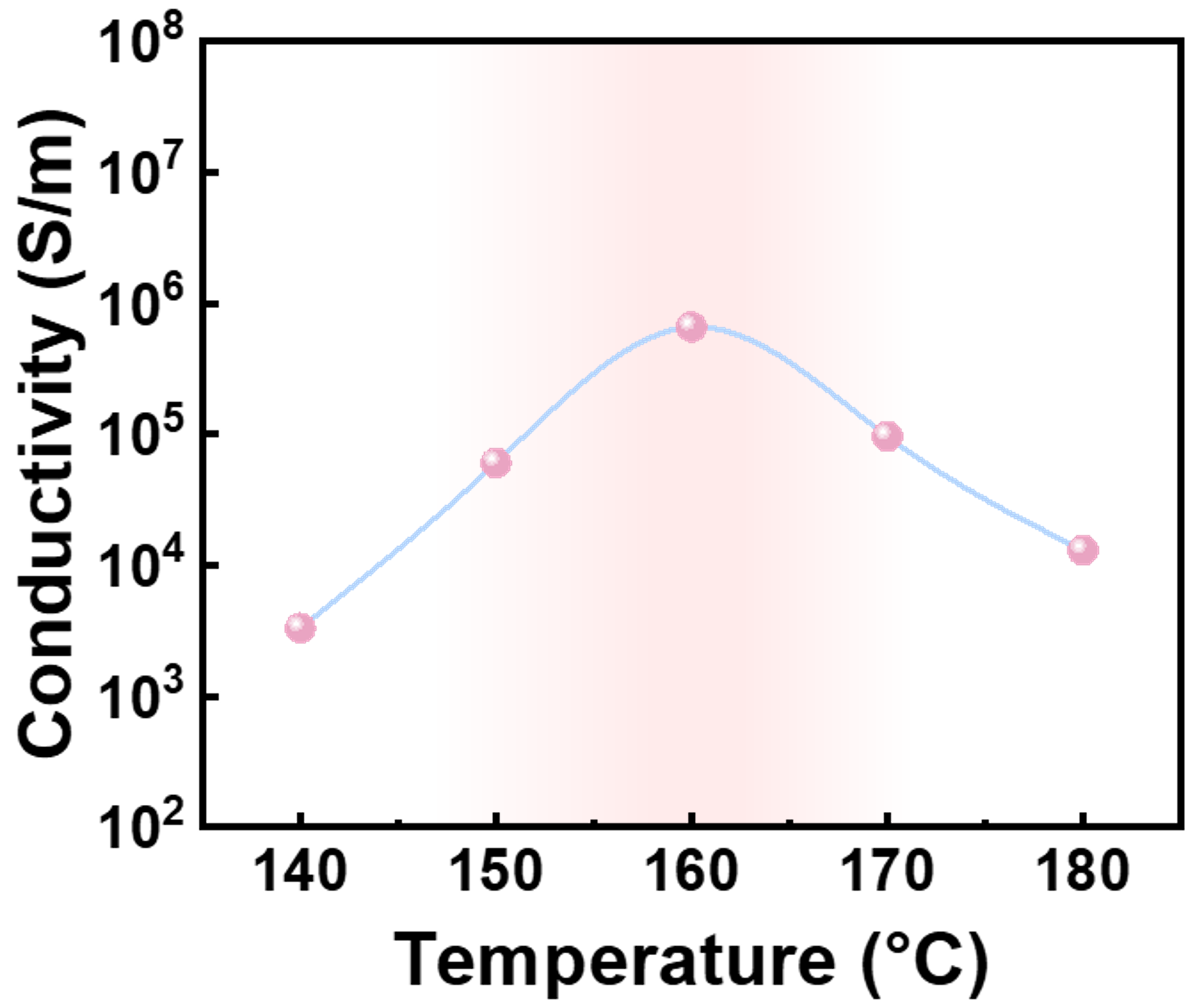
**

**Figure S25.** The conductivity of 55%-SLMPFs prepared under different processing temperatures at a fixed pressure of 0.6 MPa. The optimal processing temperature changes to 160 °C under 0.6 MPa.


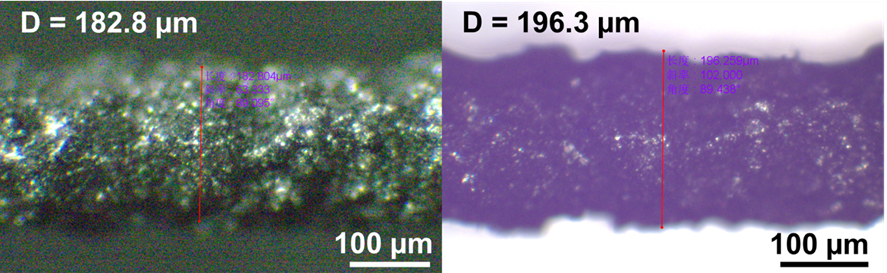


**Figure S26**. The morphologies of 55%-SLMPFs show the smallest diameters (182.8 µm and 196.3 µm) we achieved in this study. The red lines represent the measurement regions.


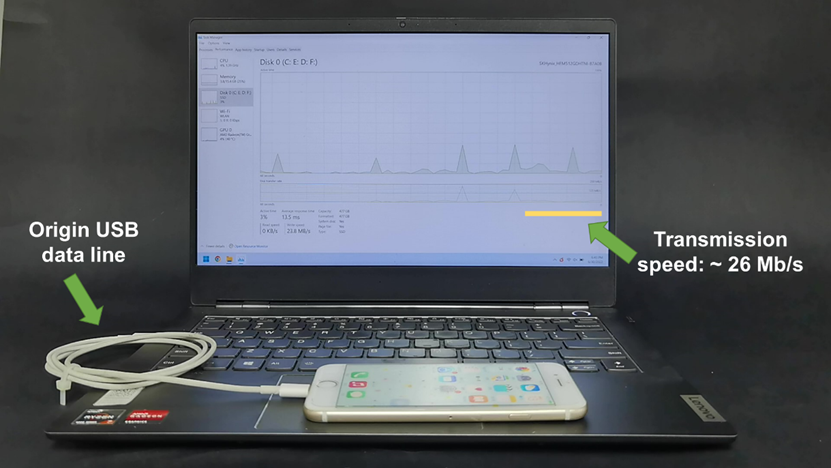


**Figure S27**. The photo shows the transmission speed (~ 26 Mb/s) between iPhone and personal computer by origin USB data line.


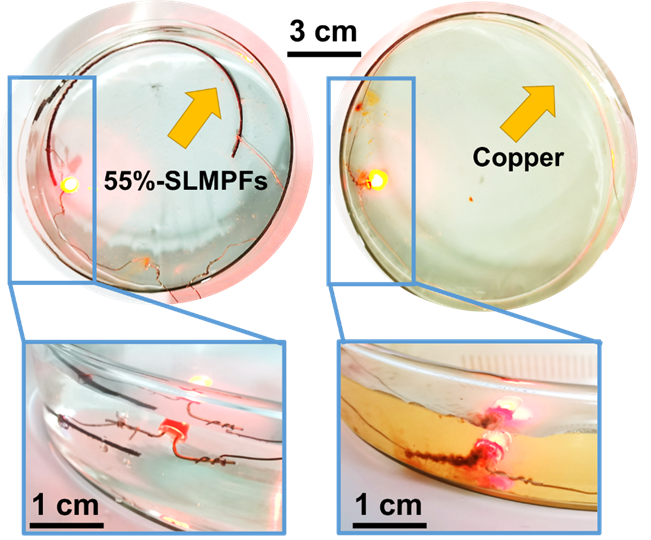


**Figure S28**. Photos of the underwater circuit made from 55%-SLMPFs (left) and commercial copper (right). After 24 h, the 55%-SLMPFs resist the corrosion while the copper circuit was corroded obviously.


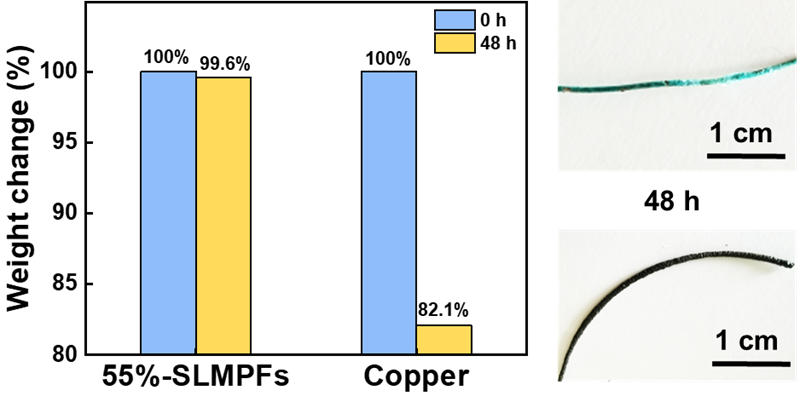


**Figure S29**. The weight change (%) of commercial copper and 55%-SLMPFs before and after power on. The copper wire was corroded obviously and turned green that its weight changed from 0.3317 to 0.2871 g. The weight of 55%-SLMPFs had negligible change (Detailed changes shown in **Figure 4f**). Inserts are photos of the underwater circuit made from commercial copper (left) and 55%-SLMPFs (right) after 48 h.

**Movie S1.** The electromagnetic induction of the coil wound by SLMPFs. Moving the magnet around the coil, a current would be induced into the coil by the physical movement of the magnetic flux inside it. The positive and negative symbols represent the direction of the induced current.

**Movie S2.** The animation process of injection simulated by Fluent software.

**Movie S3.** The mobile phone was charging by the 55%-SLMPF replaced USB data line which is no delay in charging response.

**Movie S4.** The transmission speed between iPhone and personal computer by 55%-SLMPFs USB data line.

**Movie S5.** The LED bulb was lightened by a 55%-SLMPF spiral circuit under the water whose electrical property underwater is stable.

References

[1] H. Fujita, T. Kato, "On the Navier-Stokes initial value problem, I," Arch. Rational Mech. Anal, vol. 16, no. 4, pp. 269-315, 1964.

[2] "Non-Newtonian fluids: Introduction and guide to classification and characteristics," Nasa Sti/recon Technical Report N, vol. 83, pp. 1-30, 1982.

[3] N. Phan-Thien, N. Mai-Duy, Understanding Viscoelasticity: An Introduction to Rheology (New Mexico: Springer, 2017).
